# Bayesian networks in neuroscience: a survey

**DOI:** 10.3389/fncom.2014.00131

**Published:** 2014-10-16

**Authors:** Concha Bielza, Pedro Larrañaga

**Affiliations:** Departamento de Inteligencia Artificial, Universidad Politecnica de MadridMadrid, Spain

**Keywords:** Bayesian networks, probabilistic inference, learning from data, supervised classification, association discovery, neuroimaging, connectivity analysis

## Abstract

Bayesian networks are a type of probabilistic graphical models lie at the intersection between statistics and machine learning. They have been shown to be powerful tools to encode dependence relationships among the variables of a domain under uncertainty. Thanks to their generality, Bayesian networks can accommodate continuous and discrete variables, as well as temporal processes. In this paper we review Bayesian networks and how they can be learned automatically from data by means of structure learning algorithms. Also, we examine how a user can take advantage of these networks for reasoning by exact or approximate inference algorithms that propagate the given evidence through the graphical structure. Despite their applicability in many fields, they have been little used in neuroscience, where they have focused on specific problems, like functional connectivity analysis from neuroimaging data. Here we survey key research in neuroscience where Bayesian networks have been used with different aims: discover associations between variables, perform probabilistic reasoning over the model, and classify new observations with and without supervision. The networks are learned from data of any kind–morphological, electrophysiological, -omics and neuroimaging–, thereby broadening the scope–molecular, cellular, structural, functional, cognitive and medical– of the brain aspects to be studied.

## 1. Introduction

A Bayesian network (BN) (Pearl, 1988; Koller and Friedman, 2009) is a compact representation of a probability distribution over a set of discrete variables. Variables represent the uncertain knowledge of a given domain and are depicted as the nodes of the network. The structure of a BN is a directed acyclic graph (DAG), where the arcs have a formal interpretation in terms of probabilistic conditional independence. The quantitative part of a BN is a collection of conditional probability tables, each attached to a node, expressing the probability of the variable at the node conditioned on its parents in the network. The joint probability distribution (JPD) over all variables is computed as the product of all these conditional probabilities dictated by the arcs. This distribution entails enough information to attribute a probability to any event expressed with the variables of the network. Moreover, there are efficient algorithms for computing any such probability without having to generate the underlying JPD (this would be unfeasible in many cases). BNs have enormously progressed over the last few decades leading to applications spanning all fields.

Computational neuroscience is currently an interdisciplinary science, also allied with statistics and computer science (more specifically with machine learning). Since BNs are probabilistic models, the realm of statistics offers *inference* tools to perform probabilistic reasoning under uncertainty. Machine learning algorithms are distinguished by the target outcome or the type of available input data. Thus, they have several aims: association discovery, supervised classification and clustering. BNs can support all these facilities. In *association discovery* (reviewed in Daly et al., 2011), we look for relationships among the variables of interest when we have access to data on those variables. Examples of this modeling task in neuroscience include functional connectivity analysis with fMRI or the discovery of relationships among morphological variables in dendritic trees. In *supervised classification* (reviewed in Bielza and Larrañaga, 2014) there is a discrete class (or outcome) variable that guides the learning process and which has to be predicted for new data. Sometimes there may be a vector of class variables (multi-dimensional classification). Examples in neuroscience are the classification of cortical GABAergic interneurons from their morphological or their electrophysiological characteristics or the prediction of Alzheimer's disease (AD) from the genomic-wide information. In *clustering* (reviewed in Pham and Ruz, 2009), the goal is to group the data in homogeneous groups and with a probabilistic membership assignment to each of the clusters. In neuroscience grouping dendritic spines is an example.

In this paper we try to pinpoint neuroscience problems that have been addressed using BNs. Section 2 reviews BNs and Section 3 explains how to perform inference over a BN. Section 4 describes learning algorithms used to construct the structure and estimate the probabilities that define a BN. Section 5 surveys neuroscience research using BNs, distinguishing between different input data types: morphological, electrophysiological, -omics data and neuroimaging. Section 6 rounds the paper off with a discussion.

## 2. Bayesian networks

### 2.1. Definition

BNs (Pearl, 1988; Koller and Friedman, 2009) are widely used models of uncertain knowledge. They provide a compact representation of the JPD *p*(*X*_1_, …, *X*_*n*_) across many variables **X** = (*X*_1_, …, *X*_*n*_) with values *x*_*i*_ ∈ Ω_*X*_*i*__ = {1, 2, …, *r*_*i*_}. It is the JPD over all the variables of a domain that is of great interest, since it contains all the information and can be used to ask any probabilistic question. By using the *chain rule*, the JPD can be expressed as

(1) $$p(X_{1},\ldots ,X_{n})=p(X_{1})p(X_{2}|X_{1})p(X_{3}|X_{1},X_{2})\cdots p(X_{n}|X_{1},\ldots ,X_{n-1}).$$

Note that this expression can be written in as many different ways as there are orderings of the set {*X*_1_, …, *X*_*n*_}. Despite factorization, the JPD still requires a number of values that grows exponentially with the number *n* of variables (e.g., we need 2^*n*^ − 1 values if all variables are binary). By exploiting the conditional independence between variables, we can avoid intractability by using fewer parameters and a compact expression. Two random variables *X* and *Y* are *conditionally independent* (c.i.) given another random variable *Z* if

p(x|y,z)=p(x|z) ∀x,y,z values of X,Y,Z,

that is, whenever *Z* = *z*, the information *Y* = *y* does not influence the probability of *x*. *X*, *Y*, *Z* can even be disjoint random *vectors*. The definition can be equivalently written as

p(x,y|z)=p(x|z)p(y|z) ∀x,y,z values of X,Y,Z.

Conditional independence is halfway between the intractable *complete* dependence of Equation (1) and the infrequent and unrealistic case of *mutual independence*, where *p*(*X*_1_, …, *X*_*n*_) = *p*(*X*_1_)*p*(*X*_2_)*p*(*X*_3_) … *p*(*X*_*n*_). Conditional independence is central to BNs. Suppose that we find for each *X*_*i*_ a subset **Pa**(*X*_*i*_) ⊆ {*X*_1_, …, *X*_*i*−1_} such that given **Pa**(*X*_*i*_), *X*_*i*_ is c.i. of all variables in {*X*_1_, …, *X*_*i*−1_}\ **Pa**(*X*_*i*_), i.e.,

(2) $$p(X_{i}|X_{1},\ldots ,X_{i\;-\;1})=p\left(X_{i}|Pa(X_{i})\right).$$

Then using Equation (2), the JPD in Equation (1) turns into

(3) $$p(X_{1},\ldots ,X_{n})=p\left(X_{1}|Pa(X_{1})\right)\cdots p\left(X_{n}|Pa(X_{n})\right)$$

with a (hopefully) substantially reduced number of parameters.

A BN represents this factorization of the JPD with a DAG. A *graph* 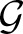 is given as a pair (*V*, *E*), where *V* is the set of nodes and *E* is the set of edges between the nodes in *V*. Nodes of a BN represent the domain random variables *X*_1_, …, *X*_*n*_. A *directed* graph has directed edges (arcs) from one node to another. Arcs of a BN represent probabilistic dependences among variables. They are quantified by conditional probability distributions shaping the interaction between the linked variables. The *parents* of a node *X*_*i*_, **Pa**(*X*_*i*_), are all the nodes pointing at *X*_*i*_. Similarly, *X*_*i*_ is their *child*. Thus, a BN has two components: a DAG and a set of conditional probability distributions of each node *X*_*i*_ given its parents, *p*(*X*_*i*_|**Pa**(*X*_*i*_)), that determine a unique JPD given by Equation (3). The first qualitative component is called the *BN structure* and the second quantitative component is called the *BN parameters*. When all the nodes are discrete variables these parameters are tabulated in what is usually referred to as conditional probability table (CPT).

**Figure 1:**
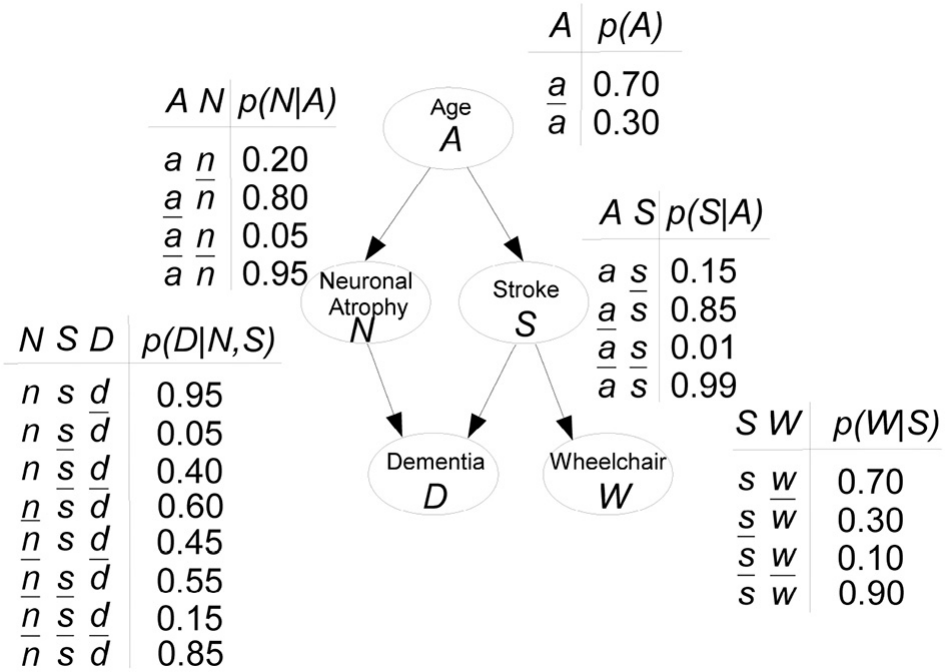
**Hypothetical example of a BN modeling the risk of dementia**.

*Hypothetical example on risk of dementia*. Figure 1 shows a hypothetical example of a BN, inspired in Burge et al. (2009), modeling the risk of dementia. All variables are binary, with *x* denoting “presence” and $\bar{x}$ denoting “absence,” for Dementia *D*, Neuronal Atrophy *N*, Stroke *S* and confined to a Wheelchair *W*. For Age *A*, *a* means “aged 65+” and otherwise the state is ā. Both Stroke and Neuronal Atrophy are influenced by Age (their parent). These two conditions influence Dementia (their child). Wheelchair is directly associated with having a stroke. Attached to each node, CPTs indicate the specific conditional probabilities. For instance, if someone has neuronal atrophy and has had a stroke, there is a 0.95 probability he will be demented: *p*(*d*|*n*, *s*) = 0.95.

The JPD is factorized as:

p(A,N,S,D,W)=p(A)p(N|A)p(S|A)p(D|N,S)p(W|S).

Thus, the JPD *p*(*A*, *N*, *S*, *D*, *W*) requires 2^5^ − 1 = 31 parameters to be fully specified. With the BN that allows the JPD factorization, only 11 input probabilities are needed.

The term *acyclic* means that the graph contains no cycles, that is, there is no sequence of nodes starting and ending at the same node by following the direction of the arcs. The *descendants* of a node *X*_*i*_ are all the nodes reachable from *X*_*i*_ by repeatedly following the arcs. Let **ND**(*X*_*i*_) denote the *non-descendants* of *X*_*i*_. The conditional independences encoded by a BN that allow to factorize the JPD as in Equation (3) are

$$X_{i}\text{ is c}.\text{i}.\text{ of }ND(X_{i})\text{ given }Pa(X_{i}),i=1,\ldots ,n,$$

that is, each node is c.i. of its non-descendants, given its parents. Then it is said that 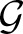 satisfies the *Markov condition* with a probability distribution *p* and that (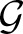, *p*) is a BN. Note that in the Dementia example, there are no cycles. The descendants of node *S* are *D* and *W*, whereas all nodes are descendants of *A*. Applying the Markov condition to node *S*, we have that *S* and *N* are c.i. given *A*.

Indeed, the Markov condition implies the factorization in Equation (3): if we simply use the chain rule Equation (1) with an ancestral (also called topological) node ordering (i.e., parents come before their children in the sequence), the non-descendants and parents will be in the conditioning sets {*X*_1_, …, *X*_*i*−1_} of the chain rule and the application of the Markov condition will give Equation (2) and hence expression (3). Conversely, given a DAG 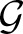 and the product in Equation (3), then the Markov condition holds. In the Dementia example, ancestral orderings are e.g., *A*-*N*-*S*-*D*-*W* or *A*-*S*-*W*-*N*-*D*.

Other conditional independences may be derived apart from those given in the Markov condition. Some may be obtained from the properties of the conditional independence relationship. But it is easier to check a property called *d-separation* over the graph which is always a sufficient condition for conditional independences in *p*. Two sets of nodes **X** and **Y** are d-separated by a third set **Z** (**X**, **Y**, **Z** are disjoint) if and only if every undirected path between **X** and **Y** is “blocked,” i.e., there is an intermediate variable *V* (not belonging to **X** or **Y**) such that: (a) *V* is a converging connection in the path, and *V* and its descendants do not belong to **Z**, or (b) *V* is not converging (serial or diverging connection) and it belongs to **Z**. A converging connection is *A* → *V* ← *B*; a serial connection is *A* → *V* → *B* or *A* ← *V* ← *B*; a diverging connection is *A* ← *V* → *B*. Thus, given the Markov condition, if node *X* is d-separated from node *Y* given node *Z*, then *X* and *Y* are c.i. given *Z*. BNs are said to be an *independence map* of *p*. If the reverse also holds, i.e., conditional independence implies d-separation (which is not always true for every distribution), then it is said that *p* is *faithful* to 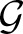 or 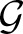 is a *perfect map* of *p*. In this case, all the independences in the distribution are read directly from the DAG.

The Markov condition is also referred to as *local Markov property*. The *global Markov property* states that each node *X*_*i*_ is c.i. of all other nodes in the network given its so-called *Markov blanket*, **MB**(*X*_*i*_), i.e.,

$$p(X_{i}|X\setminus \{X_{i}\})=p\left(X_{i}|MB(X_{i})\right).$$

If *p* is faithful to 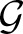, the Markov blanket of a node is composed of its parents, its children and the parents of its children (spouses). Therefore, the only knowledge required to predict the behavior of *X*_*i*_ is **MB**(*X*_*i*_). This will be relevant in supervised classification problems (Section 4.3.1).

In the Dementia example, **MB**(*N*) = {*A*, *D*, *S*}. *A* is the parent of *N*, *D* is its child and *S* is its spouse.

The 3-node BNs *X* → *Y* → *Z*, *X* ← *Y* → *Z*, and *X* ← *Y* ← *Z* are (Markov) equivalent because exactly the same conditional independences are imposed. The concept of equivalence between DAGs partitions the space of DAGs into a set of equivalence classes. This will be useful for learning BNs (see Section 4). The completed partially DAG (CPDAG) or essential graph represents all members of an equivalence class. It has an arc *X* → *Y* if it appears in every DAG belonging to the same equivalence class and otherwise has a link *X* − *Y* (either direction *X* → *Y* or *X* ← *Y* is possible in the DAGs within the equivalence class).

Arcs in a BN represent probabilistic dependences, and variables at the tails of the arcs will not necessarily be *causally* dependent on variables at the head. Arc reversals in causal relationships would change their meaning (not true in the previous equivalent BNs). In general, causality cannot be inferred from observational data alone. Data subjected to interventions are required. Differentiating between arcs needs some prior knowledge (prohibiting certain directions) or the application of external interventions that probe some arc direction using a hypothesis test. For a BN to be a causal network (Pearl, 2000), there has to be an explicit requirement for the relationships be causal. In these networks, the impact of external interventions can be predicted from data collected prior to intervention.

To sum up, a BN is a DAG and a collection of DAG-dependent conditional probability distributions whose multiplication defines the JPD (equivalently, the Markov condition holds), and, also, d-separations in the DAG imply their respective conditional independences. This modularity through the local conditional distributions makes the BN easier to maintain as there are less parameters to be estimated/elicited and stored and assures a more efficient posterior reasoning (inference).

### 2.2. Gaussian Bayesian networks

A common approach is to discretize variables *X*_1_, …, *X*_*n*_ if they are continuous, i.e., to partition them into nominal intervals. For instance, the continuous blood-oxygen-level-dependent (BOLD) responses measured by an fMRI scanner can be discretized into four categories: very low, low, high, and very high. Standard discretization methods use a fixed number *K* of equal width partitions or partitions of *K*% of the total data. Other methods in supervised classification use variable relationships to the class variable to define the bins (Fayyad and Irani, 1993). However, discretization involves some loss of information and the assignment of many parameters. Models with continuous variables are a wise choice in this case.

Unlike the categorical distributions represented by a BN, *Gaussian BNs* (Shachter and Kenley, 1989; Geiger and Heckerman, 1994) assume that the JPD for **X** = (*X*_1_, …, *X*_*n*_) is a multivariate (non-singular) normal distribution 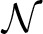
(**μ**, **Σ**), given by

(4) $$f(x)=\frac{1}{{\left(2\pi \right)}^{n/2}|\Sigma {|}^{1/2}}\exp\left(-\frac{1}{2}{\left(x-\mu \right)}^{T}\Sigma^{-1}(x-\mu )\right),$$

where **μ** = (μ_1_, …, μ_*n*_)^*T*^ is the vector of means, **Σ** is the *n* × *n* covariance matrix and |**Σ**| is its determinant. A Gaussian BN can be equivalently defined (as in (3)) as the product of *n* univariate normal densities defined as 
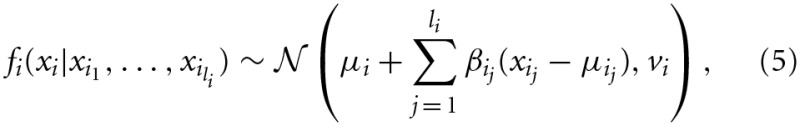
 where {*X*_i_1__, …, *X*_*i*_*l*_*i*___} = **Pa**(*X*_*i*_), μ_*i*_ is the unconditional mean of *X*_*i*_ (i.e., the *i*th component of **μ**), *v*_*i*_ is the conditional variance of *X*_*i*_ given values for *x*_*i*_1__, …, *x*_*i*_*l*_*i*___ and β_*i*_*j*__ is the linear regression coefficient of *X*_*i*_*j*__ in the regression of *X*_*i*_ on **Pa**(*X*_*i*_). It reflects the strength of the relationship between *X*_*i*_ and *X*_*i*_*j*__: there is no arc from *X*_*i*_*j*__ to *X*_*i*_ whenever β_*i*_*j*__ = 0. Note that *v*_*i*_ does not depend on the conditioning values *x*_*i*_1__, …, *x*_*i*_*l*_*i*___. Root nodes (without parents) follow unconditional Gaussians. The parameters that determine a Gaussian BN are then **μ** = (μ_1_, …, μ_*n*_)^*T*^, {*v*_1_, …, *v*_*n*_} and {β_*i*_*j*__, *i* = 1, …, *n*, *j* = 1, …, *l*_*i*_}.

*Example*. In a 4-node structure with arcs *X*_1_ → *X*_3_, *X*_2_ → *X*_3_ and *X*_2_ → *X*_4_, distributions are

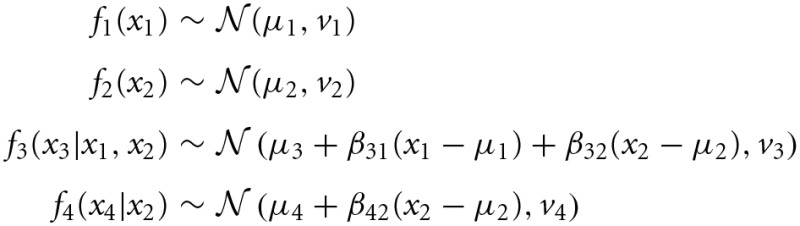



For a multivariate Gaussian density given by Equation (4), there exist formulas to generate a Gaussian BN, i.e., the product of normal densities given in Equation (5), and vice versa (Shachter and Kenley, 1989; Geiger and Heckerman, 1994). The factorized expression is better suited for model elicitation since it has to be guaranteed that the covariance matrix **Σ** is positive-definite in the multivariate expression.

Gaussian BNs assume that the interaction between variables are modeled by linear relationships with Gaussian noise. Discrete BNs are more general, able to model non-linear relationships. Strict assumptions of Gaussianity over the continuous conditional distributions in BNs can be relaxed with non-parametric density estimation techniques: kernel-based densities, mixtures of truncated exponentials (Moral et al., 2001), mixtures of polynomials (Shenoy and West, 2011) and mixtures of truncated basis functions (Langseth et al., 2012). Nevertheless, the use of these kinds of models for learning and simulation is still in its infancy, and many problems have yet to be solved.

### 2.3. Dynamic Bayesian networks

The BN models discussed so far are static. In domains that evolve over time (e.g., the sequential activation of brain areas during cognitive decision making), we need *dynamic BNs* (Dean and Kanazawa, 1989; Murphy, 2002). A discrete time-stamp is introduced and the same local model is repeated for each unit of time. That local model is a section of the network called a *time slice* and represents a snapshot of the underlying evolving temporal process. The nodes within time slice *t* can be connected to other nodes within the same slice. Also, time slices are inter-connected through temporal or transition arcs that specify how variables change from one time point to another. Temporal arcs only flow forward in time, since the state of a variable at one time point is determined by the states of a set of variables at previous time points. A prior BN specifies the initial conditions. In dynamic BNs, the structures of the time slices are identical and the conditional probabilities are also identical over time. Therefore, dynamic BNs are time-invariant models, and *dynamic* only means that they can model dynamic systems. For inference purposes, the structure of a dynamic BN is obtained by unrolling the transition network over all consecutive times.

Mathematically, a dynamic BN represents a discrete-time stochastic process where there is a vector of interest **X**^*t*^ = (*X*^*t*^_1_, …, *X*^*t*^_*n*_) at each time *t* = 1, …, *T*. For instance, the BOLD response of *n* regions of interest (ROIs) at time *t*. It is common to assume stationarity, that is, the probability does not depend on *t*. When the stochastic dynamic process is also assumed to be a first-order Markovian transition model, i.e., *p*(**X**^*t*^|**X**^*t*−1^, …, **X**^1^) = *p*(**X**^*t*^|**X**^*t* − 1^), then

$$p\left(X^{1},\ldots ,X^{T}\right)=p\left(X^{1}\right)\prod_{t\;=\;2}^{T}p\left(X^{t}|X^{t\;-\;1}\right).$$

*p*(**X**^1^) are the initial conditions, factorized according to the prior BN. *p*(**X**^*t*^|**X**^*t* − 1^) will be also factorized over individual *X*^*t*^_*i*_ as $\prod_{i\;=\;1}^{n}p\left(X_{i}^{t}|Pa^{t}(X_{i})\right)$, where **Pa**^*t*^(*X*_*i*_) may be in the same or previous time-slice. In continuous settings, a Gaussian is mostly assumed for *p*(*X*^*t*^_*i*_|**Pa**^*t*^(*X*_*i*_)) (auto-regressive model). Higher-order and non-stationary Markov models allow more complex temporal processes. However, such complex models pose obvious challenges to structure and parameter estimation.

**Figure 2:**
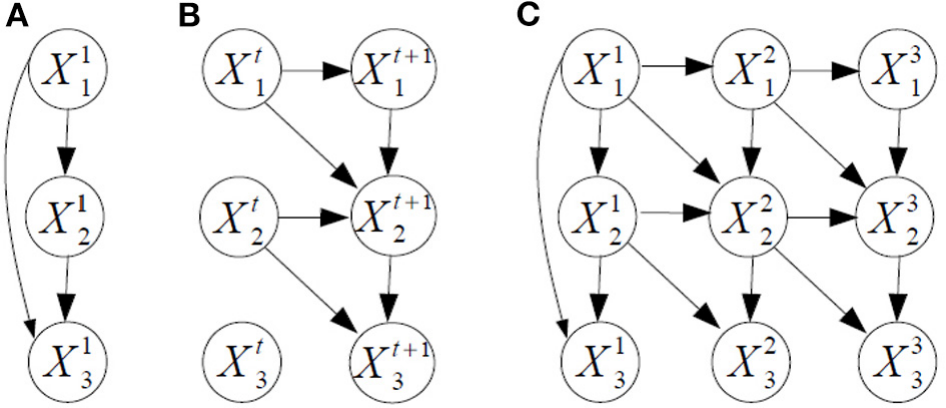
**Example of dynamic BN structure with three variables *X*_1_, *X*_2_, and *X*_3_ and three time slices. (A)** The prior network. **(B)** The transition network, with first-order Markov assumption. **(C)** The dynamic BN unfolded in time for three time slices.

*Example*. Figure 2 shows an example of dynamic BN structure. The prior and transition networks are given, respectively, in Figures 2A,B. There are three variables, *X*_1_, *X*_2_, and *X*_3_ in the problem. The two slices of nodes in Figure 2B express with temporal arcs a plate to travel from a generic time *t* to *t* + 1, in this case a first-order Markovian transition model. The order would be two if there were also arcs from *X*^*t*^_*i*_ to *X*^*t* + 2^_*i*_. For reasoning about the dynamic BN, the transition network can be unfolded in time to have a single network, see Figure 2C for *T* = 3. Note that setting arc directions across time guarantees the acyclicity of the graph, required for a BN. Dynamic BNs are able to model recurrent networks, important in neural systems as there exist cyclic functional networks in the brain, such as cortico-subcortical loops.

Dynamic BNs may assume full or partial observability of states at the nodes. For instance, neuroimaging techniques provide only indirect observations of the neural activity of a ROI, whose real state is unknown. A hidden or latent variable can model this situation. Another example is the target characters in brain- computer interfaces. Hidden Markov models (HMMs) are simple dynamic BNs used to model Markov processes that cannot be directly observed but can be indirectly estimated by state-dependent output, that is, the state is not directly visible, but the state-dependent output is. The goal is to determine the optimal sequence of states that could have produced an observed output sequence. The popular Kalman filter is a continuous-state version of HMMs.

**Figure 3:**
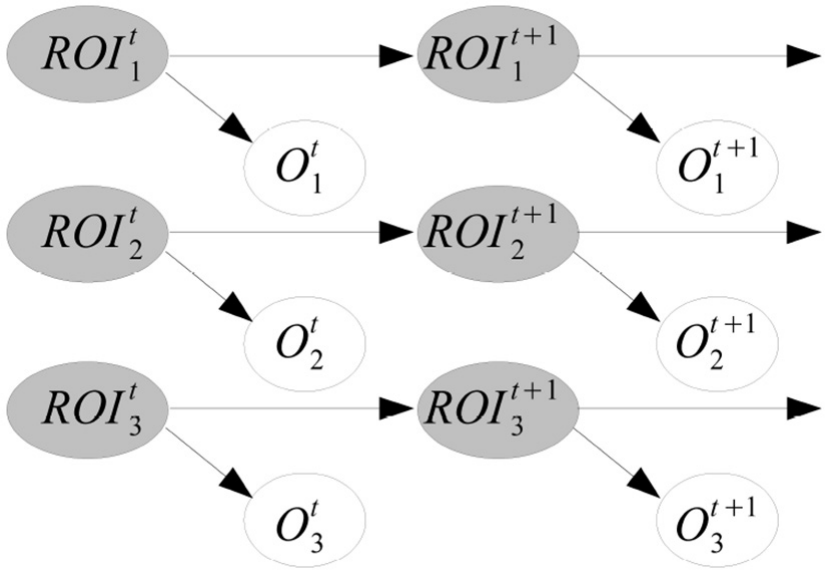
**Example of HMM.** The real state of three ROIs is unknown (gray nodes) and we indirectly observe them with BOLD responses in an fMRI experiment (white nodes).

*Example*. Figure 3 shows an example of HMM. The model represents a simple functional connectivity analysis, where 3 ROIs have been identified. Gray nodes are hidden variables and represent the unknown real neural activity of a ROI, e.g., whether the region is activated or not. White nodes are the observed measures *O*_*i*_ of each region *i*, e.g., the BOLD response in fMRI experiments. This simple model (parallel HMM) factorizes the state space into multiple independent temporal processes without connections in-between. Other versions of HMMs are more complex.

## 3. Inference with Bayesian networks

### 3.1. What is inference?

Besides visualizing the relationships between variables and deriving their conditional independences, BNs are useful for making predictions, diagnoses and explanations. To do this, the conditional probability distribution of a variable (or a set of variables) of interest is computed given the values of some other variables. The observed variables are called the *evidence* **E** = **e**. We have in **X** three kinds of variables: a query variable *X*_*i*_, the evidence variables **E** and the other, unobserved variables **U**.

Thus, inference refers to finding the probability of any variable *X*_*i*_ conditioned on **e**, i.e., *p*(*x*_*i*_|**e**). If there is no evidence, probabilities of interest are prior probabilities *p*(*x*_*i*_). Inference in BNs can combine evidence from all parts of the network and perform any kind of query. Under causality, we can predict the effect given the causes (*predictive reasoning*), diagnose the causes given the effects (*diagnostic reasoning*), explain away a cause as responsible for an effect (intercausal reasoning) or any other mixed reasoning. *Intercausal reasoning* is unique to BNs: for the v-structure *C*_1_ → *X* ← *C*_2_, *C*_1_ and *C*_2_ are independent, but once their shared child variable *X* is observed they become dependent. That is, when the effect *X* is known, the presence of one explanatory cause renders the alternative cause less likely (it is explained away).

Inference also refers to finding values of a set of variables that best explain the observed evidence. This is called *abductive inference*. In *total abduction* we solve arg max_**u**_ *p*(**u**|**e**), i.e., the aim is to find the most probable explanation (MPE), whereas the problem in *partial abduction* is the same but for a subset of variables in **u** (the explanation set), referred to as partial maximum a posteriori (MAP). These problems involve not only computing probabilities but also solving an optimization problem.

Computing these probabilities is conceptually simple, since by definition

$$p(x_{i}|e)=\frac{p(x_{i},e)}{p(e)}\propto \sum_{u}p(x_{i},e,u).$$

The term *p*(*x*_*i*_, **e**,**u**) is the JPD. It can be obtained with factorization Equation (3) which uses the information given in the BN, the conditional probabilities of each node given its parents. Using the JPD we can respond to all possible inference queries by marginalization (summing out over irrelevant variables **u**). However, summing over the JPD takes exponential time due to its exponential size, and more efficient methods have been developed. The key issue is how to exploit the factorization to avoid the exponential complexity.

**Figure 4:**
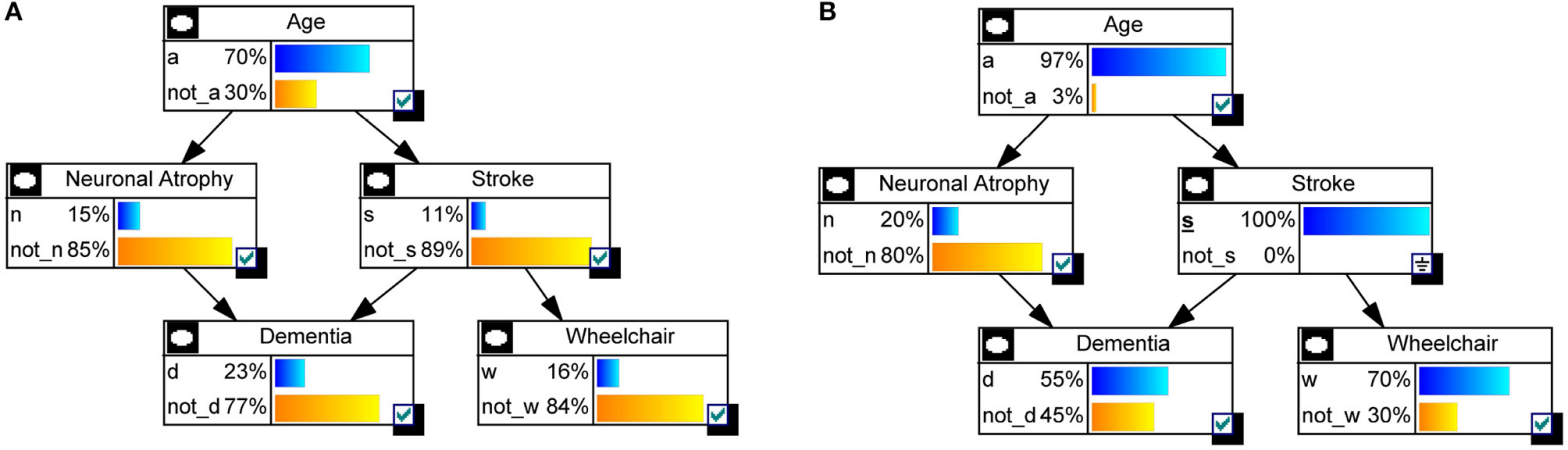
**Inference on the Dementia example. (A)** Prior probabilities *p*(*x*_*i*_) are shown as bar charts, for each node *X*_*i*_. **(B)** After observing someone who has had a stroke (*S* = *s*), the probabilities are updated as *p*(*x*_*i*_|*S* = *s*).

*Example of Dementia (continued)*. Let us take the Dementia example in Figure 1 to see how a BN is actually used. The first interesting probabilities to look at are the prior probabilities *p*(*x*_*i*_), i.e., without any evidence observed. Figure 4A shows those probabilities as bar charts. Note that the probability of being demented is 0.23. Now assume we have a patient who has had a stroke. Then the updated probabilities given this evidence, i.e., *p*(*x*_*i*_|*s*) for any state *x*_*i*_ of nodes *A*, *N*, *D*, or *W*, are shown in Figure 4B. The probability of being demented has now increased to *p*(*d*|*s*) = 0.55. However, for a patient who has not had a stroke, *p*(*d*|$\bar{s}$) = 0.19 (not shown).

### 3.2. Inference methods

*Exact BN inference* is NP-hard (Cooper, 1990) in general BNs. Therefore, an exact general algorithm to perform probabilistic inference efficiently over all classes of BNs is a long way off. On this many good special-case algorithms have been designed in order to cut down the possibly exponential time taken.

A first idea is to use the factored representation of the JPD for efficient marginalization. When summing (marginalizing) out irrelevant terms, the distributive law can be used to try to “push sums in” as far as possible.

*Example*. Suppose that we are interested in the probability of a patient having a stroke if he is not demented, *p*(*s*|$\bar{d}$). We have

$$\begin{array}{l} p(s|\bar{d})\propto \sum_{A,W,N}p(A,N,W,s,\bar{d})\\ \;=\sum_{N}p(\bar{d}|N,s)\sum_{A}p(N|A)p(s|A)p(A)\sum_{W}p(W|s). \end{array}$$

Note the use of the distributive law.

The query values (small letter) are always fixed and the unobserved nodes (capitals) are varied. All the functions that contain an unobserved variable are multiplied before marginalizing out the variable. The innermost sums create new terms which then need to be summed over. The summation order could have been different. This algorithm is called *variable elimination*.

Other algorithms operate over restricted BN structures, like *polytrees*. Polytrees are DAGs with no loops, irrespective of arc direction. They are also called *singly-connected BNs*, since any two nodes are linked with only one path. There are exact algorithms that can perform efficient and local inference on polytrees in polynomial time, the most important being the *message-passing* algorithm (Pearl, 1988). Each node acts here as an autonomous processor that collects messages (information) from its family (parents and children), performs processing and sends back messages to its family. Unlike the variable elimination algorithm that has to be run for every target node possibly repeating many computations, the posterior probabilities of all variables, i.e., *p*(*x*_*i*_|**e**) for all *X*_*i*_ not in the evidence set **E**, are computed with the message-passing algorithm in twice the time it takes to compute the posterior probability of a single variable.

*Multiply-connected BNs* contain at least one pair of nodes connected by more than one path. The Dementia network is an example. The message-passing algorithm is not directly applicable because the messages can loop forever. A popular solution is called the *clustering approach* (Lauritzen and Spiegelhalter, 1988). It consists of transforming the BN structure into an alternative graph with a polytree structure, called *junction tree*, by appropriately merging or clustering some variables to remove the multiple paths between two nodes. Thus, the nodes in the junction tree can include several variables. A message-passing algorithm is then run over the junction tree.

In Gaussian BNs, inference is easy since any conditional distribution is still Gaussian and the updated parameters, mean and variance, have closed formulas (Lauritzen and Jensen, 2001; Cowell, 2005). In general BNs with non-parametric density estimation techniques, inference has been performed only on networks with a small number of nodes (Cobb and Shenoy, 2006; Rumí and Salmerón, 2007; Shenoy and West, 2011).

For general networks, non-standard distributions and many nodes, we need to resort to approximate inference. *Approximate inference* in general BNs is also NP-hard (Dagum and Luby, 1993). Many stochastic simulation techniques are based on Monte Carlo methods, where we use the network to generate a large number of cases (full instantiations) from the JPD, and then the probability is estimated by counting observed frequencies in the samples. A well-known method is *probabilistic logic sampling* (Henrion, 1988). Given an ancestral ordering of the nodes, we generate from a node once we have generated from its parents (forward sampling scheme). Other techniques are *Gibbs sampling* and more general Markov chain Monte Carlo (MCMC) methods. In Gibbs sampling we generate samples from the distribution of *X*_*i*_ conditioned on all current values of the other nodes at each step. This distribution only involves the CPTs of the Markov blanket of *X*_*i*_ (thanks to the global Markov property) (Pearl, 1988). After judging the convergence of the underlying Markov chain, whose stationary distribution is the JPD, the values simulated for each node are a sample generated from the target distribution. Evidence variables are fixed rather than sampled during the simulation.

## 4. Learning Bayesian networks from data

The structure and conditional probabilities necessary for characterizing the BN can be provided either externally by experts–time consuming and error prone– or by automatic learning from data. This is the approach taken in this section. The learning task can be separated into two subtasks (Section 2.1): *structure learning* and *parametric learning*.

### 4.1. Learning Bayesian network parameters

There are two main approaches: (a) *maximum likelihood estimation*, where the estimation of the parameters maximize the likelihood of the data, resulting in relative frequencies for multinomial data and in sample mean and sample variance for Gaussian BNs; and (b) *Bayesian estimation*, where the prior distributions are usually chosen to be conjugate with respect to multinomial (Dirichlet distribution) or Gaussian (Wishart density) distributions (Spiegelhalter and Lauritzen, 1990; Geiger and Heckerman, 1997). The maximum likelihood approach has problems with sparse data because some conditional probabilities can be undefined if the data set does not contain all possible combinations of the involved variables. To avoid this, some form of prior distribution is usually placed on the variables, which is then updated from the data.

One important problem in learning BNs is to deal with missing data, a problem that occurs in most real-life data sets. In the context of missing at random, where the missing mechanism depends on the observed data, the most widely used method of parameter estimation is the expectation maximization (EM) algorithm (Dempster et al., 1977), first applied in BNs by Lauritzen (1995).

### 4.2. Learning Bayesian network structures. associations

Although almost all methods are designed for multiply-connected BNs, there are some proposals where the topology of the resulting network is reduced to trees or polytrees. One algorithm that recovers a *tree structured BN*, that is, a structure where each node has one parent (except the root node), is based on work by Chow and Liu (1968). Their algorithm constructs the optimal second-order approximation to a JPD by finding the maximum weighted spanning tree, where each branch is weighted according to the mutual information between the corresponding two variables. The first approach for learning polytrees from data was proposed by Rebane and Pearl (1987).

**Figure 5:**
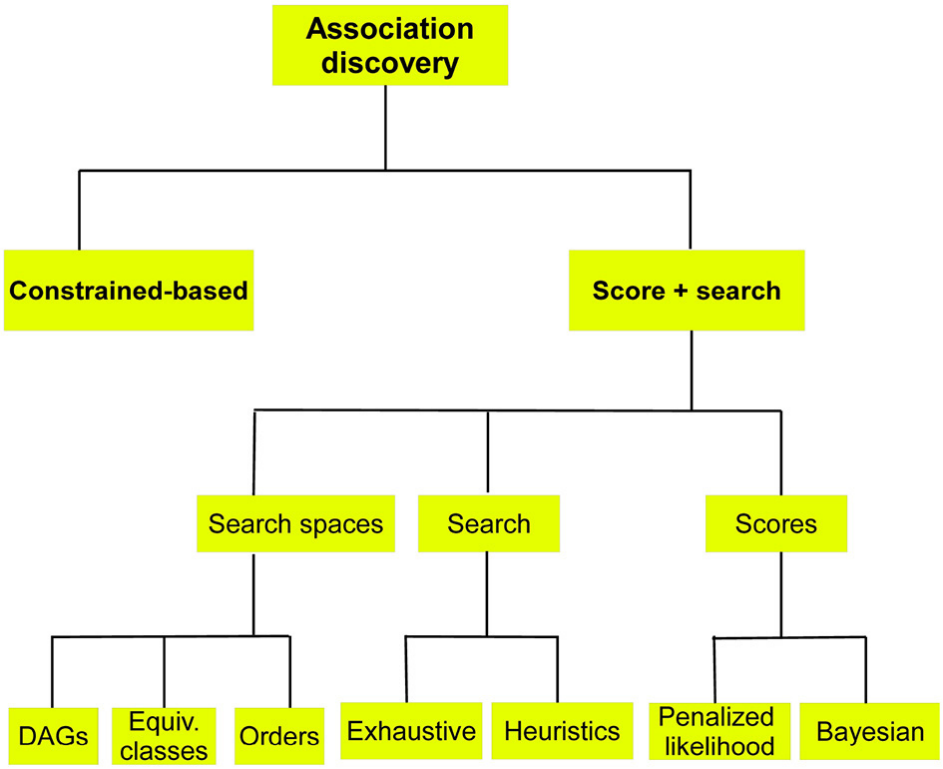
**Schematic of methods for BN structure learning when the aim is the discovery of associations among variables**.

Two approaches are discussed next, see Figure 5.

#### 4.2.1. Constrained-based methods

Learning BNs by means of constrained-based methods means that conditional independences among triplets of variables are statistically tested from data and a DAG that represents a large percentage (and whenever possible all) of these conditional independence relations is then drawn.

The *PC algorithm* (Spirtes and Glymour, 1991), where PC stands for “Peter and Clark,” the first names of the two inventors of the method, starts by assuming that all nodes in the undirected graph are connected and uses hypothesis tests to delete connections. At each iteration, for each node *X* and each node *Y* adjacent to *X*, sets of nodes adjacent to *X* (excluding *Y*) are searched in order to find a conditioning set that renders *X* and *Y* c.i. The edge between *X* and *Y* is removed if and only if this set is found. At each iteration of the PC algorithm, the number in the conditioning set increases. Note that if the cardinality of the conditioning sets increases, the statistical test for checking conditional independences reduces its reliability. The undirected graph is then transformed into a CPDAG by means of some orientation rules. Some variants and extensions of the PC algorithm include a limitation on the number of conditional independence tests (Margaritis and Thrun, 2000), the control of the false positive rate (Li and Wang, 2009), and the extension of the PC algorithm in the Gaussian BN context with conditional independence tests based on sample partial correlations (Kalisch and Bühlmann, 2007).

#### 4.2.2. Score and search methods

In these methods a score measuring the goodness of each candidate BN is computed. Candidate BNs are proposed using a search method responsible for intelligent movements in the space of possible structures. Three different spaces can be considered: (a) the space of DAGs; (b) the space of Markov equivalent classes; and (c) the space of orderings, see Figure 5.

The *space of DAGs* has a cardinality that is super-exponential in the number of nodes (Robinson, 1977). The problem of finding the best BN structure according to some score from the set of all networks in which each node has no more than *K* parents (*K* > 1) is NP-complete (Chickering, 1996). This offers a chance to use different *heuristic search* algorithms. Almost all types of heuristics have been applied for structure learning, including greedy search (Buntine, 1991; Cooper and Herskovits, 1992), simulated annealing (Heckerman et al., 1995), genetic algorithms (Larrañaga et al., 1996b), MCMC methods (Friedman and Koller, 2003) and estimation of distribution algorithms (Blanco et al., 2003).

*The space of Markov equivalent classes* is a reduced version of the space of DAGs where all Markov equivalent DAGs are represented by a unique structure (Section 2.1). Working in this new space avoids the movements between DAGs within the same equivalence class thereby reducing the cardinality of the search space. Gillispie and Perlman (2002) found that the ratio of DAGs to numbers of classes is seemingly close to an asymptote of about 3.7. A drawback of working in this space is that it is time consuming to check whether or not a structure belongs to the same equivalence class. A seminal paper on using equivalence classes is Chickering (2002), whereas extensions include its randomized version (Nielsen et al., 2003) and an adaptation to Gaussian BNs (Vidaurre et al., 2010).

*The space of orderings* is justified by the fact that some learning algorithms only work with a fixed order of variables, assuming that only the variables that precede a given variable, can be its parents. This assumption dramatically reduces the cardinality of the search to *n*!. Seminal works include Singh and Valtorta (1993) using conditional independence tests, Bouckaert (1992) who manipulates the ordering of the variables with operations similar to arc reversals, Larrañaga et al. (1996a) with a genetic algorithm-based search, and Romero et al. (2004) using estimation of distribution algorithms.

*Scores* measure the goodness of fit of a BN structure to the data set (the better the fit, the higher the score). One simple criterion able to measure this fit is the *log-likelihood of the data given the BN*. This can be expressed as

(6) $$\log p(D|S,\;\theta )=\sum_{i\;=\;1}^{n}\sum_{j\;=\;1}^{q_{i}}\sum_{k\;=\;1}^{r_{i}}N_{ijk}\log(\theta_{ijk}),$$

where *D* denotes the data set containing *N* cases, *S* represents the structure of the BN, **θ** is the vector of parameters θ_*ijk*_, and *N*_*ijk*_ stands for the number of cases in *D* where variable *X*_*i*_ is equal to *k* and **Pa**_*i*_ is in its *j*-th value, *j* = 1, …, *q*_*i*_. The maximum likelihood estimator of θ_*ijk*_ is given by its relative frequency, that is, ${\hat{\theta }}_{ijk}=\frac{N_{ijk}}{N_{ij}}$, where $N_{ij}=\sum_{k\;=\;1}^{r_{i}}N_{ijk}$. A drawback of using likelihood as the score is that it increases monotonically with the complexity of the model, and, as consequence of this property, the structure that maximizes the likelihood coincides with the complete graph. A family of *penalized log-likelihood* scores has been proposed as an alternative that aims to find a trade-off between fit and complexity. Their general expression is

(7) $$\sum_{i\;=\;1}^{n}\sum_{j\;=\;1}^{q_{i}}\sum_{k\;=\;1}^{r_{i}}N_{ijk}\log\frac{N_{ijk}}{N_{ij}}-dim(S)pen(N),$$

where $dim(S)=\sum_{i\;=\;1}^{n}q_{i}(r_{i}-1)$ denotes the model dimension (number of parameters necessary to specify the structure), and *pen*(*N*) is a non-negative penalization function. The scores are different depending on *pen*(*N*): if *pen*(*N*) = 1, the score is called *Akaike's information criterion* (Akaike, 1974) and when *pen*(*N*) = $\frac{1}{2}$ log *N*, it is the Bayesian information criterion (BIC) (Schwarz, 1978).

A Bayesian approach attempts to find the structure with maximum a posteriori probability given the data, that is, arg *max*_*S*_*p*(*S*|*D*). Using Bayes' formula, *p*(*S*|*D*) ∝ *p*(*D*|*S*)*p*(*S*), where *p*(*D*|*S*) is the *marginal likelihood* of the data, defined as

p(D|S)=∫p(D|S,θ)p(θ|S)dθ,

and *p*(*S*) denotes the *prior distribution* over structures. Assuming that all structures are equally likely, that is, *p*(*S*) is uniform, the maximization of *p*(*S*|*D*) is equivalent to the maximization of the marginal likelihood.

With the additional assumption of a uniform distribution for *p*(**θ**|*S*), Cooper and Herskovits (1992) were able to find a closed formula for the marginal likelihood

(8) $$p(D|S)=\prod_{i\;=\;1}^{n}\prod_{j\;=\;1}^{q_{i}}\frac{(r_{i}-1)!}{(N_{ij}+r_{i}-1)!}\prod_{k\;=\;1}^{r_{i}}N_{ijk}!.$$

This is called the *K2 score*.

Similarly, assuming a uniform distribution for *p*(*S*) and a Dirichlet distribution with parameters α_*ijk*_ for *p*(**θ**|*S*), Heckerman et al. (1995) obtained the following expression for the marginal likelihood

(9) $$p(D|S)=\prod_{i\;=\;1}^{n}\prod_{j\;=\;1}^{q_{i}}\frac{\Gamma (\alpha_{ij})}{\Gamma (\alpha_{ij}+N_{ij})}\prod_{k\;=\;1}^{r_{i}}\frac{\Gamma (\alpha_{ijk}+N_{ijk})}{\Gamma (\alpha_{ijk})},$$

where Γ denotes the Gamma function, and $\alpha_{ij}=\sum_{k\;=\;1}^{r_{i}}\alpha_{ijk}$. This score is called the Bayesian Dirichlet equivalence with uniform prior (BDeu) metric because it verifies the score equivalence property (two Markov equivalent graphs score equally) and is generally applicable when the search is carried out in the space of equivalence classes. Geiger and Heckerman (1994) adapted the BDeu score to Gaussian BNs.

Learning from data the first-order Markovian dynamic BNs presented in Section 2.3 can be approached by adapting either of both types of methods (constrained-based or score and search). The prior network structure can be learned from a data set containing samples at time *t* = 1, whereas the transition network can be recovered from a data set composed by samples from times *t* − 1 and *t*, with *t* = 1, 2, …, *T*. This last data set includes 2*n* variables.

### 4.3. Learning Bayesian network structures. supervised classification

Given a data set of labeled instances, *D* = {(**x**^1^, *c*^1^), …, (**x**^*N*^, *c*^*N*^)}, the *supervised classification model* (or simply the classifier) denoted by ϕ transforms points from the instance space Ω**_X_** into points in the label space Ω_*C*_, that is,

$$\begin{array}{l} \;\Omega_{X}\overset{\phi }{\to }\Omega_{C}\\ x=(x_{1},\ldots ,x_{n})\to \phi (x) \end{array}$$

The *i*-th component of **x**, *x*_*i*_, contains the value of the *i*-th predictor variable, *X*_*i*_, for one specific instance. BN classifiers perform classification selecting the class value c^*^ such that

(10) $$c^{*}=\arg\underset{c}{\max}p(c|x)=\arg\underset{c}{\max}p(x,c).$$

With a zero-one loss this rule coincides with the Bayes decision rule.

Although there is a large set of supervised classification models (Hastie et al., 2008), some of which are probabilistic classifiers (Murphy, 2012), the use of Bayesian classifiers has many advantages over other classification techniques. From a representation point of view, they are BNs thereby having the same advantages (Section 1). From the algorithm perspective: (a) algorithms that learn Bayesian classifiers from data are computationally efficient, with a learning time complexity that is linear on the number of cases *N*, and linear, quadratic or cubic (depending on model complexity) on the number of variables *n*; and (b) classification time is linear on the number of variables *n*.

Neuroscience is a field where the volume of available data is starting to grow exponentially, especially data produced by neuroimaging, sensor-based applications and innovative neurotechnologies, like extracellular electrical recording, optimal imaging, ultrasound and molecular recording devices. In such situations, *feature subset selection* methods should be used to delete irrelevant and redundant variables from the set of predictors, and have definite benefits (Saeys et al., 2007), such as: (a) reduction of the computation time in both learning and classification processes; and (b) simpler models providing insight into the problem. We will see below that only a small percentage of the revised papers incorporated this dimensionality reduction possibility.

Once the Bayesian classifier has been learned from data, the model will be used to predict the class value of new instances, which are each characterized by their predictor variables only. One interesting issue is to measure the goodness of the model. Several *performance measures* are in use (Japkowicz and Mohak, 2011), including accuracy, error rate, sensitivity, specificity, positive predictive value, negative predictive value, *F* measure, Cohen's kappa statistics, Brier score, total cost error, and the area under the receiver operating characteristic curve (AUC). In neuroscience, the systematic use of accuracy and AUC is noteworthy, with very few references to the other performance measures. Another aspect to be considered is how to properly estimate the selected performance measures. Estimates cannot be calculated on the same data set used for learning the classifier, because the aim is to estimate the goodness of the model on new instances. An honest method estimates the value of the performance measure based on samples that have not previously been seen in the learning phase by the classifier. Hold-out and *k*-fold cross-validation are representative of single resampling methods, while repeated hold-out, repeated *k*-fold cross-validation, bootstrap and randomization are examples of multiple resampling. Interestingly, not all revised papers related to supervised classification consider honest estimation. For the papers where this circumstance was taken into account, *k*-fold cross-validation was the preferred method.

#### 4.3.1. Discrete Bayesian network classifiers

**Figure 6:**
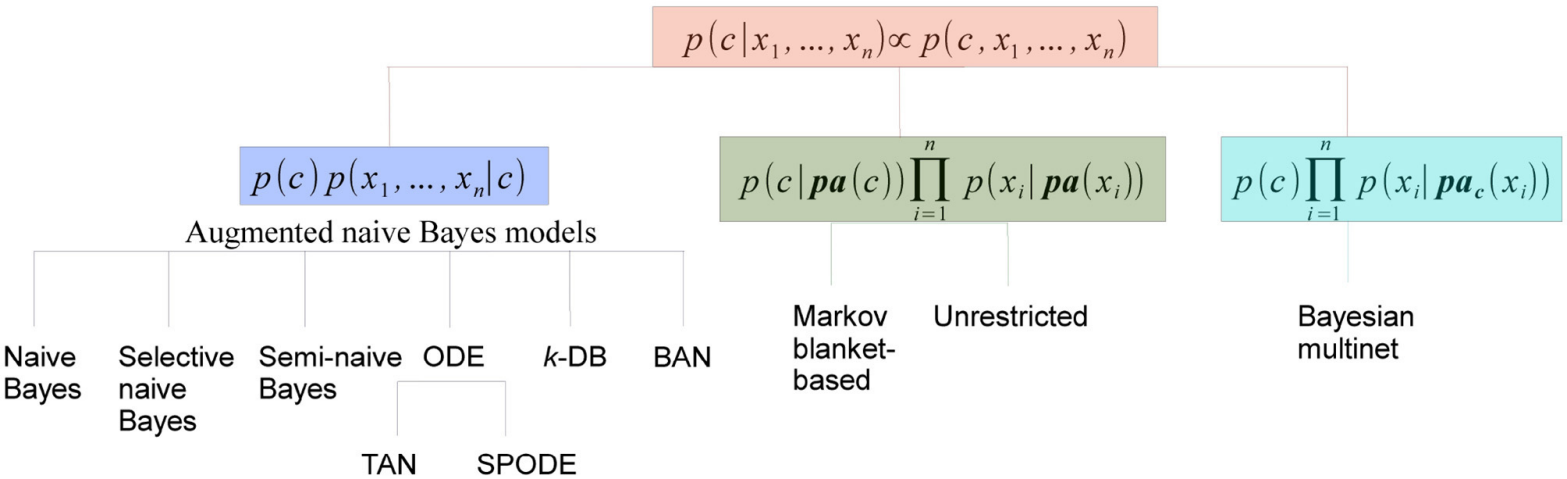
**Categorization of discrete BN classifiers according to the factorization of *p*(**x**, *c*)**.

Discrete BN classifiers, reviewed in Bielza and Larrañaga, (2014), contain discrete variables as predictors. *p*(*c*|**x**) is computed considering that *p*(*c*|**x**) ∝ *p*(**x**, *c*) and factorizing *p*(**x**, *c*) according to a BN structure. The different families of discrete Bayesian network classifiers are in fact the result of the different manners of factorizing *p*(**x**, *c*), as shown in Figure 6. We will consider three families: (a) augmented naive Bayes, (b) classifiers where *C* has parents, and (c) Bayesian multinets. In this review we have only found neuroscience applications of naive Bayes, selective naive Bayes, tree-augmented naive Bayes, *k*-dependence Bayesian classifiers, unrestricted Bayesian classifier and Bayesian multinet. The discussion below will focus on these models.

*(a) Augmented naive Bayes* models cover some discrete Bayesian classifiers characterized by: (i) *C* being the parent of all predictor variables and having no parents; and (ii) the level of dependence among predictor variables increasing gradually.

*Naive Bayes* (Maron and Kuhns, 1960) is the simplest BN classifier. It assumes that predictive variables are c.i. given the class, resulting in

(11) $$p(c|x)\propto p(c)\prod_{i\;=\;1}^{n}p(x_{i}|c).$$

An example of a naive Bayes structure is given in the first row of Table 1. In this case, the conditional probability of the class variables is computed as *p*(*c*|**x**) ∝ *p*(*c*)*p*(*x*_1_|*c*)*p*(*x*_2_|*c*)*p*(*x*_3_|*c*)*p*(*x*_4_|*c*)*p*(*x*_5_|*c*). Although naive Bayes assumptions of conditional independences do not hold in real-world applications, its model classification performance may still be good from a practical point of view, especially when *n* is high and/or *N* is small. Both situations apply in neuroscience applications, and this partly justifies the widespread use of naive Bayes in the reviewed papers, as confirmed in Tables 2–**6**.

**Table 1:**
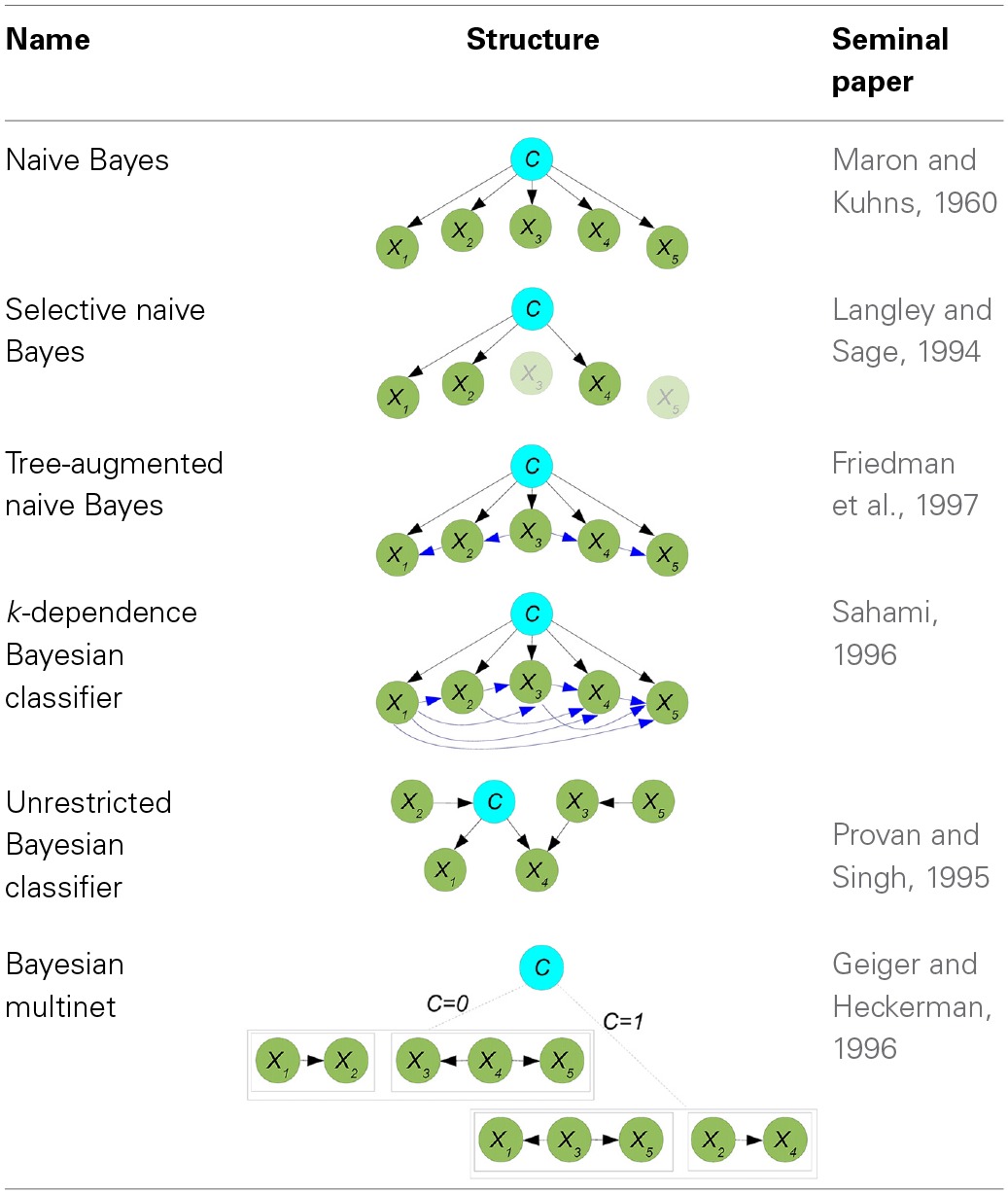
**Summary of discrete BN classifiers: names, structures and their most relevant reference**.

**Table 2:** **Main characteristics of the papers using BNs with morphological data**.

	**BN model**	**Aim**	**Application**
DeFelipe et al., 2013	BN and naive Bayes	Assoc. and supervised class	Classification and naming of GABAergic interneurons
Lopez-Cruz et al., 2014	BN and BN multinet	Assoc. and inference and cluster	Consensus model for interneuron classification
Mihaljevic et al., in press	Naive Bayes and TAN	Supervised class	Classification of cortical GABAergic interneurons
Mihaljevic et al., Under review	MBC	Multi-dimensional class	Simultaneous classification of six axonal class variables
Guerra et al., 2011	Naive Bayes	Supervised class	Pyramidal neuron vs. interneuron
Lopez-Cruz et al., 2011	BN	Inference, associations	Model and simulation of dendritic trees

*Selective naive Bayes* (Langley and Sage, 1994) aims at considering relevant and non-redundant predictor variables. The selection process reduces the cost of the acquisition of the data and, at the same time, improves the performance of the model. The conditional probability of the class variables is now computed as

(12) $$p(c|x)\propto p(c|x_{F})=p(c)\prod_{i\;\in \;F}p(x_{i}|c),$$

where **X**_*F*_ denotes the set of selected predictors. The second row of Table 1 shows a selective naive Bayes structure where shaded variables have not been selected, and the conditional probability of *C* is calculated as *p*(*c*|**x**) ∝ *p*(*c*)*p*(*x*_1_|*c*)*p*(*x*_2_|*c*)*p*(*x*_4_|*c*). An extension of naive Bayes that considers all possible naive Bayes structures which are then averaged in a single model was defined in Dash and Cooper (2004) as *model-averaged naive Bayes* and applied in neuroscience as shown in **Table 4**.

The *semi-naive Bayes* (Pazzani, 1996) model modifies the initial conditional independence assumption of naive Bayes by introducing new variables obtained as the Cartesian product of two or more original predictor variables.

One-dependence Bayesian classifiers (ODEs) are Bayesian classifiers where each predictor variable is allowed to depend on at most another predictor in addition to the class. We will consider two ODEs: tree-augmented naive Bayes and superparent-one-dependence estimators. Tree-augmented naive Bayes (TAN) (Friedman et al., 1997) violates the conditional independence condition allowing a tree shape graph as the subgraph representing the relationships among predictor variables. The conditional distribution of *C* is now

(13) $$p(c|x)\propto p(c)p(x_{r}|c)\prod_{i\;=\;1,i\;\neq \;r}^{n}p(x_{i}|c,x_{j(i)}),$$

where *X*_*r*_ denotes the root node and {*X*_*j*(*i*)_} = **Pa**(*X*_*i*_)\ {*C*}, for any *i* ≠ *r*. Kruskal's algorithm (Kruskal, 1956) is used to find a maximum weighted spanning tree among predictor variables, where the weight of each edge is measured by the conditional mutual information between each pair of variables given the class. The undirected tree is then transformed into a directed tree by selecting a variable at random as the root node and then converting the edges into arcs accordingly. Finally, a naive Bayes structure is superimposed on the tree in order to obtain the TAN structure. The third row of Table 1 contains a TAN structure with *X*_3_ as the root node. Classification is performed using *p*(*c*|**x**) ∝ *p*(*c*)*p*(*x*_1_|*c*, *x*_2_)*p*(*x*_2_|*c*, *x*_3_)*p*(*x*_3_|*c*)*p*(*x*_4_|*c*, *x*_3_)*p*(*x*_5_|*c*, *x*_4_). An example of the use of a TAN classifier in the prediction of brain metastasis is shown in Table 2. Superparent-one-dependence estimators (SPODEs) (Keogh and Pazzani, 2002) are ODEs where in addition to the class all predictor variables depend on the same predictor variable, called the superparent.

*k*-dependence Bayesian classifiers (*k*-DB) (Sahami, 1996) allows each predictor variable to have a maximum of *k* parent variables apart from the class variable. Naive Bayes and TAN are particular cases of *k*-DBs, with *k* = 0 and *k* = 1, respectively. The conditional probability distribution of *C* is

(14) $$p(c|x)\propto p(c)\prod_{i\;=\;1}^{n}p(x_{i}|c,x_{i_{1}},\ldots ,x_{i_{k}}),$$

where *X*_*i*_1__, …, *X*_*i*_**k**__ are the *k* parents of *X*_*i*_ in the structure. An example of a 3-DB structure from which *p*(*c*|**x**) ∝ *p*(*c*)*p*(*x*_1_|*c*)*p*(*x*_2_|*c*, *x*_1_)*p*(*x*_3_|*c*, *x*_1_, *x*_2_)*p*(*x*_4_|*c*, *x*_1_, *x*_2_, *x*_3_)*p*(*x*_5_|*c*, *x*_1_, *x*_3_, *x*_4_) is shown in the fourth row of Table 1. The robustness of the estimation of the probabilities of the last factor in the above expression can be problematic with small sample sizes. The parents of each predictor variable are determined by computing the conditional mutual information between any pair of predictor variables given the class (as in TAN) and also the mutual information between this predictor variable and the class.

Finally, Bayesian network-augmented naive Bayes (BAN) (Ezawa and Norton, 1996) uses any BN structure as the predictor subgraph, allowing any kind of relationship among predictor variables.

*(b) Classifiers where *C* has parents* provide conditional probability distributions of *C* of the form

(15) $$p(c|x)\propto p(c|pa(c))\prod_{i\;=\;1}^{n}p\left(x_{i}|pa(x_{i})\right).$$

The two types of models in this family differ on whether or not *C* is considered as a special variable. *Markov blanket-based Bayesian classifiers* (Koller and Sahami, 1996) consider *C* as a special variable and the Bayesian classifier is based on identifying the Markov blanket of *C*. *Unrestricted Bayesian classifiers* do not consider *C* as a special variable in the induction process, where any existing BN structure learning algorithm can be used. The corresponding Markov blanket of *C* can be used later for classification purposes. The fifth row of Table 1 contains one example providing the same conditional distribution as the previous example. This type of classifiers have been used in **Tables 5**, **6**.

*(c) Bayesian multinets* (Geiger and Heckerman, 1996) are able to encode asymmetric conditional independences, that is, conditional independence relationships that only hold for some, but not all, the values of the variables involved. They consist of several local BNs associated with a domain partition provided by a distinguished variable. For supervised classification problems, the class variable usually plays the role of distinguished variable. Thus, conditioned on each *c*, the conditional independences among predictor variables can be different.

Bayesian multinets compute the conditional probability of the class variable as

(16) $$p(c|x)\propto p(c)\prod_{i\;=\;1}^{n}p\left(x_{i}|pa_{c}(x_{i})\right),$$

where **Pa**_*c*_(*X*_*i*_) is the parent set of *X*_*i*_ in the local BN associated with *C* = *c*.

If the number *N* of observations is small, the decision about the class label is usually made by averaging the results (or even the models themselves) provided by several classification models. This constitutes an *ensemble of Bayesian classifiers*. Examples can be seen in **Tables 4**, **5**.

More challenging classification problems consider the simultaneous prediction of several class variables that are related to each other. This is called *multi-dimensional classification*. An example of this situation is the classification of GABAergic interneurons based on axonal arborization patterns (Table 2). Multi-dimensional BN classifiers (MBC) (Bielza et al., 2011) were designed to solve arg max_*c*_1_, …, *c*_*d*__ *p*(*c*_1_, …, *c*_*d*_|*x*_1_, …, *x*_*n*_) for *d* class variables.

#### 4.3.2. Continuous Bayesian network classifiers

Predictor variables can be continuous as opposed to discrete. In the first case, a common assumption is the Gaussianity of the predictors, although BN classifiers not based on this assumption have also been proposed in the literature.

*(a) Gaussian predictors. Gaussian naive Bayes classifier* (Friedman et al., 1998) assumes that the conditional density of each predictor variable, *X*_*i*_, given a value of the class variable, *c*, follows a Gaussian distribution, that is, *X*_*i*_|*C* = *c* ~ 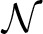
(μ_*i*, *c*_, σ_*i*, *c*_) for all *i* = 1, …, *n*, *c* ∈ Ω_*C*_. For each *c*, parameters μ and σ have to be estimated. Maximum likelihood is usually the estimation method. This model has been extensively applied in neuroscience problems (see **Tables 5**, **6**). Pérez et al. (2006) show adaptations of other discrete BN classifiers to Gaussian predictors.

*(b) Non-Gaussian predictors. Kernel-based BN classifiers* estimate the conditional densities of predictor variables by means of kernels. The so-called *flexible naive Bayes classifier* (John and Langley, 1995) was the first proposal, later extended to *flexible TAN* and *flexible *k*-DB classifiers* by Pérez et al. (2009) (see an example in **Table 6**).

### 4.4. Learning Bayesian networks structures. clustering

The main goal of clustering (Jain et al., 1999) is to find the natural grouping of the data. Clustering methods can be organized as non-probabilistic (mainly, hierarchical clustering Ward, 1963 and *k*-means MacQueen, 1967) or probabilistic, only the latter being related to BNs.

*Probabilistic clustering* assumes the existence of a hidden (latent) variable containing the cluster assignment to each object. The different methods are commonly based on *Gaussian mixture models* (Day, 1969), where a mixture of several Gaussian distributions is used to adjust the density of the sample data when the fitting provided by a single density is not good enough. The probability density function in a Gaussian mixture model is defined as a weighted sum of *K* Gaussian densities

$$g(x|\theta )=\sum_{k\;=\;1}^{K}\pi_{k}f(x|\theta_{k}),$$

where π_*k*_ is the weight of component *k*, 0 < π_*k*_ < 1 for all components, $\sum_{k\;=\;1}^{K}\pi_{k}=1$, and *f*(**x**|**θ**_*k*_) denotes a 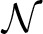
(**μ**_*k*_, **Σ**_*k*_) density. The parameter **θ** = (π_1_, **μ**_1_, **Σ**_1_, …, π_*K*_, **μ**_*K*_, **Σ**_*K*_) defines a particular Gaussian mixture model and is usually estimated with the expectation-maximization algorithm (EM) (Dempster et al., 1977). When the multivariate Gaussian density is factorized according to a Gaussian BN structure, probabilistic clustering is carried out with a probabilistic graphical model.

The simplest probabilistic graphical model for clustering is a Gaussian mixture model where each component of the mixture factorizes according to a naive Bayes model. This was proposed by Cheeseman et al. (1988) and extended to a Bayesian model averaging of naive Bayes for clustering in Santafé et al. (2006). Seminaive Bayes and Bayesian multinets for clustering were introduced by Peña et al. (1999) and Peña et al. (2002), respectively. This application of the EM algorithm for the estimation of the parameters in the mixture model with Gaussian BNs as components assumes fixed structures in each of the components. Friedman (1998) proposed a more flexible approach allowing the structures to be updated at each iteration of the EM–the so-called *structural EM*.

## 5. Bayesian networks in neuroscience

### 5.1. Morphological data

Table 2 summarizes the content of this section.

The problem of classifying and naming GABAergic interneurons has been a controversial topic since the days of Santiago Ramón y Cajal. DeFelipe et al. (2013) proposed a pragmatic alternative to this problem based on axonal arborization patterns. They described six axonal variables: (1) distribution of the interneuron axonal arborization relative to cortical layers; (2) distribution of the axonal arborization relative to the size of cortical columns; (3) relative location of the axonal and dendritic arbors; (4) distribution of the main part of the cortical surface; (5) interneuron types: common type, horse-tail, chandelier, Martinotti, common basket, large basket, Cajal-Rezius, neurogliaform and other; (6) whether or not the number of morphological axonal characteristics visualized for a given interneuron were sufficient. A web-based interactive system was used to collect data about the terminological choices on the above six variables for 320 cortical interneurons by 42 experts in the field. A BN was learned from the data of each expert maximizing the K2 score with a greedy search strategy. A set of morphological variables were extracted and used as predictor variables to automatically build BN classifiers to discriminate among the interneuron classes. To capture the opinions of all experts, Lopez-Cruz et al. (2014) developed a consensus model in the form of a Bayesian multinet. The idea was to cluster all JPDs, each related to the BN built for each expert. The Bayesian multinet encoded a finite mixture of BNs with the cluster variable as the distinguished variable. Differences were identified between the groups of experts by computing the marginal (or prior) probabilities in the representative BN for each cluster.

Instead of assigning to each neuron the interneuron class most commonly selected by the experts (majority vote), Mihaljevic et al. (in press) set different label reliability thresholds (i.e., every cell's label is supported by at least a certain number of experts), and classification models were built for each threshold. Mihaljevic et al. (Under review) simultaneously classified the six axonal class variables, with the morphological variables playing the role of predictor variables. The six-dimensional JPD can be represented by a BN that is learned from data given by the 42 experts. The six-dimensional prediction for a new neuron, can be made by the consensus BN of its *k*-nearest neurons (in the predictor variable space).

Discriminating between pyramidal cells and interneurons from mouse neocortex was proposed by Guerra et al. (2011). Neurons were reconstructed using Neurolucida and morphological variables were measured. The label of each neuron was assigned defining “ground truth” by the presence or absence of an apical dendrite.

BNs have been used to model and simulate dendritic trees from layer III pyramidal neurons from different regions (motor M2, somatosensory S2 and lateral visual and association temporal V2L/TeA) of the mouse neocortex (Lopez-Cruz et al., 2011). A set of variables were measured for each dendritic tree, providing information about the subtree and subdendrite, segment length, orientation, and bifurcation. The BN learning algorithm was based on the BIC score with a greedy search. A simulation algorithm was also proposed to obtain virtual dendrites by sampling from the BNs.

### 5.2. Electrophysiological data

Table 3 summarizes the content of this section.

**Table 3:** **Main characteristics of the papers using BNs with electrophysiological data**.

	**BN model**	**Aim**	**Application**
Smith et al., 2006	Dynamic BN	Association	Infer non-linear neural information flow networks
Eldawlatly et al., 2010	Dynamic BN	Association	Infer effective and time-varying connectivity between spiking cortical neurons
Jung et al., 2010	BN	Association	Neuronal synchrony from electrode signal recordings
Pecevski et al., 2011	BN	Inference	Emulate probabilistic inference through networks of spiking neurons

Various methods (clustering and pairwise measures) have been used to infer functional synchrony between neuronal channels using electrode signal recordings. However, these approaches fail to consider high-order and non-linear interactions, which can be recovered using BNs.

Smith et al. (2006) used dynamic BNs for inferring non-linear neural information flow networks from electrophysiological data collected with microelectrode arrays. While neural connectivity networks describe the existence of anatomical connections between different brain regions, they contain no information about which paths are utilized during processing or learning tasks undertaken by the brain. To understand these phenomena, we need flow networks. The dynamic BN with appropriately chosen sampling intervals successfully inferred neural information flow networks that matched known anatomy from electrophysiology data collected from the auditory pathway of awake, freely moving songbirds. Each of the bird had fluorescently labeled microelectrodes, each represented as a node in the dynamic BN and contained the multi-unit activity recorded using discretized values of the original voltages. A Bayesian scoring metric and a greedy search procedure with random restarts were applied.

Eldawlatly et al. (2010) used dynamic BNs to infer the effective and time-varying connectivity between spiking cortical neurons from their observed spike trains. The model assigned a binary variable to each neuron whose values depended on the neuron's firing states at a given Markov lag. This Markov lag can be adjusted considering the expected maximum synaptic latency in the pool of connections and can be seen as the model order, a measure of its complexity.

Non-dynamic BNs based on the concept of degree of combinatorial synchrony were proposed by Jung et al. (2010). Each neuronal channel was represented as a variable in the BN structure, and synchrony between neuronal channels was described by arcs. Each variable in the network contained the number of spikes per single time epoch. The time-delayed co-firing of different neuronal channels could be included in large bins of the same time epoch. The process of inferring synchrony between neuronal channels was seen as identifying neuronal connections that are highly likely to be connected in the BN structure. The BDeu score was used to measure the goodness of each candidate structure.

Pecevski et al. (2011) presented theoretical analyses and computer simulations demonstrating that networks of spiking neurons can emulate probabilistic inference for general BNs representing any JPD. The probabilistic inference was carried out from an MCMC sampling of spiking neuron networks. This result depicts probabilistic inference in BNs as a computational paradigm to understand the computational organization of networks of neurons in the brain.

### 5.3. Genomics, proteomics, and transcriptomics

Table 4 summarizes the content of this section.

**Table 4:** **Main characteristics of the papers using BNs with genomics, proteomics, and transcriptomics data**.

	**BN model**	**Aim**	**Application**
Armañanzas et al., 2012	Ensemble of BN classifiers	Association	Transcripts in AD
Hullam et al., 2012	BN	Association	SNPs in depression
Zeng et al., 2013	BN	Association	Cytokines and mRNA in cerebral ischemia
Liang et al., 2007	BN	Association	SNPs in childhood absence epilepsy
Zhang et al., 2010	BN	Association	Regulation network of the neuron-specific factor Nova (mice)
Jiang et al., 2011	BN	Association	SNPs in late onset AD
Han et al., 2012	BN	Association	SNPs in early onset autism
Wei et al., 2011	Model-averaged naive Bayes, selective naive Bayes	Sup. classification	Prediction of AD from SNPs
Gollapalli et al., 2012	Selective naive Bayes	Sup. classification	Mass spectrometry for predicting glioblastoma
Belgard et al., 2011	Naive Bayes	Sup. classification	Distinguish sequenced transcriptomes among layers I-VIb

*A. Association discovery*. Armañanzas et al. (2012) analyzed high-throughput AD transcript profiling with an ensemble of BN classifiers. The data came from a few AD and control brain samples. The aim was to understand dysregulation in the hippocampal entorhinal cortex, as well as its comparison with dentate gyrus. A resampling method with a feature selection technique and a Bayesian *k*-DB produced a gene interaction network formed by arcs above a fixed confidence level.

Hullam et al. (2012) used BNs to approximate the effect of a single nucleotide polymorphism (SNP) in the HTR1A gene on depression. Other nodes of the BNs measured recent negative life events, childhood adversity score and gender. The BN model was learned guided by a Bayesian score. Liang et al. (2007) conducted SNP studies to investigate the relationship between the CACNA1H gene and childhood absence epilepsy. Both single locus and haplotype analyses were carried out with a BN learned with a Bayesian metric guided by a greedy search.

The clinical features of cerebral ischemia and the plasma levels of the cytokines and their mRNA levels in leucocytes formed a BN in Zeng et al. (2013) to analyze causal relationships among the pro-inflammatory cytokine proteins and their mRNA counterparts. The BN was learned using L1-regularization and the BIC score. Zhang et al. (2010) proposed using BNs to identify a number of splicing events directly regulated by the neuron-specific factor Nova in the mouse brain. The BN integrated RNA-binding data, splicing microarray data, Nova-binding motifs, and evolutionary signatures.

Jiang et al. (2011) identified gene-gene interactions in a genome-wide association study using a late onset AD (LOAD) data set. The data set contained information about more than 300,000 SNPs and one binary genetic variable representing the apolipoprotein E gene carrier status. After filtering the most relevant SNPs, BNs with 1, 2, 3, and 4 parents were scored with a Bayesian metric. Han et al. (2012) addressed the same problem of characterizing SNP-disease associations using BNs with the LOAD and an autism data set. A new information-based score, designed to cope with small samples, was introduced with a branch-and-bound search method to recover the structure of the BN.

*B. Supervised classification*. Wei et al. (2011) also analyzed the LOAD data set with several types of BN classifiers. Gollapalli et al. (2012) introduced a comparative analysis of serum proteome of glioblastoma multiforme patients and healthy subjects to identify potential protein markers. Sequenced transcriptomes of different areas (primary and secondary) of the adult mouse somatosensory cortex were used as predictor variables in a naive Bayes model for distinguishing among layers I-VIb in Belgard et al. (2011).

### 5.4. Neuroimaging data

Neuroimaging is the predominant technique in cognitive neuroscience, with an increasing number of publications. The different imaging techniques vary in anatomical coverage, temporal sampling and imaged hemodynamic properties. The physical mechanisms of signal generation vary and lead to differences in signal properties. Therefore, the studies must specify the modality used. We split this section according to the following modalities: fMRI, MRI, EEG, others and multimodal mechanisms. Tabular summaries are Table 6 (fMRI and MRI) and Table 5 (other techniques).

**Table 5:** **Main characteristics of the reviewed papers that use BNs with neuroimaging data: fMRI and MRI**.

**Techniques**	**BN model**	**Aim**	**Application**
**fMRI**			
Iyer et al., 2013	Gaussian BNs	Association	Resting-state (normal subjects)
Dawson et al., 2013	Gaussian BNs	Association	Resting-state (normal subjects)
Li et al., 2011	Gaussian BNs	Association	Resting-state (normal subjects)
Li et al., 2013	Gaussian BNs	Association	Resting-state (aMCI vs. controls)
Labatut et al., 2004	Gaussian dynamic BNs	Association	Phoneme task (normal vs. dyslexic)
Li et al., 2008	Gaussian dynamic BNs	Association	Bulb squeeze (healthy vs. Parkinsonian)
Kim et al., 2008	Discretized dynamic BNs	Association	Auditory task (schizophrenia vs. controls)
Zhang et al., 2005	Mixed dynamic BNs (HMMs)	Association and sup. classification	Monetary reward task (drug addicted vs. healthy)
Rajapakse and Zhou, 2007	Discretized dynamic BNs	Association	Silent reading and counting Stroop (normal subjects)
Sun et al., 2012	Gaussian BNs	Association	Watching videos (normal subjects)
Neumann et al., 2010	CPDAGs	Association	Meta-analysis
Mitchell et al., 2004	Gaussian naive Bayes	Sup. classification	Prediction of cognitive states
Raizada and Lee, 2013	Gaussian naive Bayes	Sup. classification	Distinction of phoneme sounds
Ku et al., 2008	Gaussian naive Bayes	Sup. classification	Prediction of which category a monkey is viewing
Douglas et al., 2011	Naive Bayes	Sup. classification	Belief vs. disbelief states
Burge et al., 2009	Discretized dynamic BNs	Association and Sup. classification	Healthy vs. demented elderly subjects
Chen and Herskovits, 2007	Inverse-tree classifier	Sup. classification	Young vs. non-demented vs. demented older
	Naive Bayes (latent variable)	clustering	Inference of ROI state
**MRI**			
Joshi et al., 2010	Gaussian BN	Association	Relationships between cortical surface areas
Wang et al., 2013	Gaussian BN	Association	Interaction graphs for AD patients and controls
Chen et al., 2012a	Discretized dynamic BNs	Association	Temporal interactions in normal aging and MCI
Duering et al., 2013	Gaussian BN	Association	Processing speed deficits in VCI patients
Morales et al., 2013	Naive Bayes, selective naive Bayes	Sup. classification	Early diagnosis of Parkinson's disease
Diciotti et al., 2012	Naive Bayes	Sup. classification	Early diagnosis of AD
Zhang et al., 2014	Naive Bayes	Sup. classification	MCI vs. AD
Chen et al., 2012b	Ensemble of BNs	Sup. classification	Conversion from MCI to Alzheimer

**Table 6:** **Main characteristics of the reviewed papers that use BNs with neuroimaging data: EEG, other and multimodalities**.

**Techniques**	**BN model**	**Aim**	**Application**
**EEG**			
Song et al., 2009	Time-varying dynamic BNs	Association	Motor imagination task
De la Fuente et al., 2011	BN	Association	Borderline personality disorder
Valenti et al., 2006	Kernel naive Bayes	Sup. classification	Detection of interictal spikes (epilepsy)
Acharya et al., 2011	Naive Bayes	Sup. classification	Normal/interictal/ictal (epileptic) signals
Rezaei et al., 2006	HMM	Sup. classification	Classification of mental states
Speier et al., 2012	Gaussian naive Bayes	Sup. classification	P300 Speller (virtual keyboard)
Speier et al., 2014	HMM	Sup. classification	P300 Speller (virtual keyboard)
Zhang et al., 2006	BN	Sup. classification and inference	Hearing assessment
Hausfeld et al., 2012	Gaussian naive Bayes	Sup. classification	Speech sound identification (speakers and vowels)
De Vico Fallani et al., 2011	Gaussian naive Bayes	Sup. classification	Person identification (resting-state)
**OTHERS**			
Wang et al., 2011	Gaussian naive Bayes	Sup. classification	Distinction of semantic categories (epilepsy)
Goker et al., 2012	Gaussian naive Bayes	Sup. classification	JME vs. healthy.
Lu et al., 2014	Gaussian selective naive Bayes	Sup. classification	Mental states (activation vs. rest)
Dyrba et al., 2013	Gaussian selective naive Bayes	Sup. classification	AD vs. controls
Ayhan et al., 2013	Gaussian selective naive Bayes	Sup. classification	Levels of dementia in AD
Huang et al., 2011	Sparse Gaussian BN	Association	Resting-state (AD vs. controls)
**MULTIMODAL**			
Plis et al., 2010	Continuous dynamic BNs	Association	Integrated analysis of fMRI and MEG in one subject
Plis et al., 2011	Discretized dynamic BNs	Association	Non-repeated and repeated images with sounds
Svolos et al., 2013	Naive Bayes	Sup. classification	Atypical meningiomas vs. glioblastomas vs. metastases
Chen et al., 2013	General BN	Sup. classification	Glioblastomas vs. brain metastases
Tsolaki et al., 2013	Naive Bayes	Sup. classification	Glioblastomas vs. metastases
Turner et al., 2013	Naive Bayes	Multi-label class	Meta-analysis

#### 5.4.1. fMRI

An fMRI experiment will produce a sequence of 3D images, where in each voxel (candidate features) we have a time series of BOLD signals sampled according to the temporal resolution. This data is extremely high dimensional (millions of data observations), sparse (a few training examples), temporal and noisy posing machine learning challenges and demanding the design of feature selection and classifier training methods.

*A. Association discovery*. fMRI data are mainly used for functional connectivity analysis, which studies how different parts of the brain are integrated during the execution of sensory or cognitive tasks (with some stimuli), in a resting state (no stimuli) and/or when suffering from some neurological disease.

Three well-known methods, structural equation modeling (SEM) (McIntosh and Gonzalez-Lima, 1994), dynamic causal modeling (Friston et al., 2003), and Granger causality mapping (Goebel et al., 2003), require a prior connectivity model and are traditionally used for graphs with few nodes. The prior model is often subject to anatomical constraints and obtained in studies of monkeys, a problem for higher-order functions unique to human like language and cognition. This prior knowledge is not required for BNs, which have become an established approach in brain connectivity analysis. The power of BNs is that they can represent any multivariate probabilistic association (linear or non-linear) among discrete variables. BNs can also handle more nodes. The nodes represent the activated brain regions. A connection (arrow) between two regions represents an interaction between them, characterized by conditional probabilities. For instance, the arc from *X* to *Y* means that the activation of region *Y* statistically depends on the activation of region *X*. The brain regions are expected to collectively and interactively perform the particular cognitive task (if any) in the fMRI experiment. Thus, BNs offer a complete statistical description of network behavior, unlike SEM, for example, which provides only a second-order statistical model (covariances) of the underlying neural system. Both direct and indirect connections can be distinguished. *Indirect connections* represent how one node generates its connectivity with other node through mediating variables. Note that conditional independence between regions does not encode connectivity as directed information flow via direct or indirect anatomical links.

There are different methods of selecting ROIs. They can be selected in a data-driven way, where multiple time series are grouped according to some criterion, such as independent component analysis (ICA) (McKeown et al., 1998) based on the spatiotemporal characteristics of the BOLD signal of every single voxel. An alternative method is to select multiple ROIs from a previous analysis of the fMRI data (activation map, with the parts of brain that are active during a condition of interest) or from an anatomical brain atlas. Both methods have been used with BNs, although the atlas is more natural (a ROI is a node). The BOLD signal of a ROI is commonly taken as the average BOLD signals across all voxels inside the ROI.

The presence of multiple participating subjects in the fMRI experiment calls for group studies, where not only is it important to extract a representative brain for the group but also to consider the variability within the group. We can assume that the whole group has the same brain network and BOLD time series from each individual are concatenated and treated as sampled from a single subject. This is appropriate for small and homogeneous samples. Unfortunately, it can result in statistical dependences (arcs in the BN) that do not exist in any of the individuals (the Yule-Simpson paradox). Alternatively, we can learn a different network for each individual and then perform group analysis on the individual networks. This is appropriate for large and heterogeneous samples. An intermediate approach considers the same brain network for a group (same BN structure) but different patterns of connectivity for each individual (different parameters). These three approaches are, respectively, called virtual-typical-subject, individual-structure and common-structure in Li et al. (2008).

The directionality of connectivity should be interpreted with caution (see Section 2.1). This is an important much debated topic and warrants future research. The literature makes a distinction between the location of connections between brain regions—functional connectivity—and the direction of these connections—directional functional connectivity or effective connectivity. The major works are Smith et al. (2011), criticizing BNs for not performing well at identifying the directionality of connections in simulated single-subject data (extended to group analysis in Iyer et al., 2013), and Mumford and Ramsey (2014), who provide a solution to overcome the above failures. Generally, this primer on BNs for fMRI stresses, apart from some incorrect preprocessing steps in Smith et al. (2011), that only approaches specifically designed for fMRI data should be used. The specificities of fMRI data have triggered new BN structure learning algorithms (reviewed in Mumford and Ramsey, 2014). For instance, an extension of Greedy Equivalence Search (GES) to groups of samples is the Independent Multiple-sample GES (iMaGES) algorithm (Ramsey et al., 2010), which follows a common-structure approach. This algorithm does not concatenate the data of each individual; it uses the BIC score of the graph for each individual and combines all scores into a group score.

The BN models used in this context are Gaussian, discrete, static and dynamic BNs. Learning algorithms that rely on data Gaussianity (such as GES with the BIC score, iMaGES and the most common PC) accurately identify connections but not correct orientation. This contrasts with non-Gaussian methods, therefore recommended by Mumford and Ramsey (2014). Non-Gaussianity is more realistic for modeling fMRI data disturbances. For dynamic BNs, the inter-scan interval of fMRI is used as the time slice. Because of the computational burden of dynamic BNs, activations of brain regions have to be assumed to be stationary and follow a Markovian condition. This is less realistic because activations are constantly influenced by external/internal stimuli.

We distinguish between resting-state fMRI and task-based fMRI. In Iyer et al. (2013) still normal subjects give rise to a Gaussian BN (actually networks or constellations of regions; one was the default mode network), learned using the PC algorithm. The algorithm was applied to averaged-across-subjects connection matrices, i.e., c.i. tests were applied over averaged correlation matrices. A similar idea was followed in Dawson et al. (2013) for the visual cortex. Gaussian BNs were again used in Li et al. (2011) for subjects with their eyes closed. ICA was first applied to identify the nodes used. Candidate BNs were scored with BIC. Only significant connections given by a hypothesis test of the regression coefficients were kept in this work. Negative connections were interpreted as competitive relations between sensory and cognitive processing. Resting-state fMRI is also applied to medical research. Patients with amnestic MCI (aMCI), the prodromal stage of AD, and controls under the resting condition were studied by Li et al. (2013). ROIs only covered the default mode network, whose abnormal functioning is associated with AD.

Functional connectivity analysis is harder in fMRI experiments where stimuli are present, because of design complexity and variability. Labatut et al. (2004) explored normal vs. dyslexic subjects during phoneme categorization tasks. Li et al. (2008) modeled fMRI data from healthy and Parkinson's disease patients, all asked to squeeze a rubber bulb. Dynamic BN structures were sampled with MCMC and then averaged according to their appearance frequencies. A different BIC score was defined for structure learning depending on the group-analysis approach. Distinct connectivity patterns were found in Kim et al. (2008) for paranoid schizophrenia patients and controls performing an auditory oddball task.

The two groups in Zhang et al. (2005) were drug-addicted and healthy subjects. The aim was to study the loss of sensitivity to the relative value of money in cocaine users. Hidden variables were introduced in an HMM because the state of each region is considered unknown and the only observations are of activation. The observed activation of each region was modeled as a mixture of multivariate (a vector of voxels) Gaussians conditioned by their discrete parent hidden node (activated or not activated region). Dynamic BNs were learned using the BIC score and a modified structural EM algorithm.

Rajapakse and Zhou (2007) conducted two experiments with normal subjects: silent reading of words appearing on the screen, and neutral and interference conditions in a counting Stroop task (Bush et al., 2006). Differences in the networks of each condition were explored. The learning of dynamic BNs used a Bayesian score and MCMC, where new network structures were generated by elementary operations such as deleting, adding or reversing an edge. Intra-scan connections were not allowed (since the effect on a region has a time delay). The effective connectivity of the regions was taken as the transition network connectivity. Human subjects watching videos of the same semantic category (sports, weather, advertisements) participated in Sun et al. (2012). Gaussian BNs were tested with different learning algorithms (PC, GES and IMaGES).

Neumann et al. (2010) took a different approach. Motivated by the large sample size required to get reliable BNs from their simulations, they performed a meta-analysis including several thousand activation coordinates (Talairach space) from more than 500 fMRI papers (on several experimental tasks). The BN was learned with a Bayesian score and MCMC and was then converted into a CPDAG as proposed by Chickering (2002).

*B. Supervised classification*. Within the study of cognitive processes, the prediction of cognitive state (class) given voxel activities (features) can be set out as a supervised classification problem. Mitchell et al. (2004) used a Gaussian naive Bayes classifier with different filter feature selection methods to predict the probability *p*(*c*|**x**) of cognitive state *c*, given fMRI observation **x**. Three case studies were designed to distinguish whether the subject is examining a sentence or a picture, an ambiguous or an unambiguous sentence, and the semantic categories a word belongs to. The same models were used in Raizada and Lee (2013) to produce smooth single-subject images in multivoxel pattern-based fMRI studies, i.e., the so-called searchlight analyses, where a number is written into each voxel which measures the classification in that voxel's local neighborhood. The application was to distinguish phoneme sounds. Monkeys were presented with gray-scaled images of the same category in Ku et al. (2008). Gaussian naive Bayes classifiers were also used to distinguish pairs of categories and infer which category the monkey was viewing. Feature (i.e., voxel) selection was applied using a priori knowledge: only voxels from the inferotemporal cortex and whose activation level was above a certain significance level. Douglas et al. (2011) asked subjects to evaluate the truth content of propositional statements indicating whether or not they believed it to be true. A naive Bayes was used. ICA was first performed on each subject's data set (a matrix of voxels by time points) to reduce dimensionality.

Discriminating groups of patients based on their fMRI activation patterns is again a supervised classification problem. Thus, Burge et al. (2009) used discrete dynamic BNs to identify functional correlations among ROIs in healthy and demented (AD type) elderly subjects during a visual-motor response task. The BDe score guided the structure search. Two dynamic BNs, one for each class, were learned and their differences were analyzed. The absence of a link does not necessarily mean that the link's parent and child are statistically independent. It means that there are other links corresponding to stronger relationships, as measured by the BDe score. Both networks were also to distinguish between healthy aging and dementia: the class was predicted by the model with the highest posterior likelihood. Gaussian naive Bayes was used as a competitor in this classification problem. Pairwise discrimination between young subjects, non-demented older and demented older subjects based on fMRI data from a visual-motor task was presented in Chen and Herskovits (2007). They used a special BN classifier with an inverse-tree structure, i.e., containing only arcs from each ROI node to the class node. ROIs were selected via a greedy forward search. To infer the state *R* of each ROI, they used a model with a naive Bayes structure where *R* is a latent variable, parent of all nodes, these nodes being the representative voxel for this ROI.

#### 5.4.2. MRI

*A. Association discovery*. BN modeling has been applied to find statistical brain connectivity relationships between brain areas of controls and patients with neuropathological findings.

Joshi et al. (2010) studied the dependences on cortical surface areas from gray matter measurements in normal people. Regions (nodes) for both left and right hemispheres were considered in the Gaussian BN model. The PC algorithm was applied to continuous variables following a log-normal distribution. Wang et al. (2013) performed a Gaussian BN analysis based on regional gray matter volumes to identify differences between AD patients and normal controls (NC) in structural interactions among ROIs. A score (BIC) + search approach was used to recover the BN structures.

Chen et al. (2012a) proposed dynamic BNs to model a longitudinal study of normal aging controls and MCI patients. The model assumed a discrete-time stochastic Markovian process of order one. No intra-slice arcs were assumed. The subjects were prospectively followed annually for up to 10 years. The study focused on modeling temporal interactions among some brain regions. For each region the regional gray matter was calculated and associated with the value of the corresponding node in the dynamic BN. A score + search approach was applied to learn these two dynamic BNs.

Processing speed deficits for patients with vascular cognitive impairment (VCI) was investigated with Gaussian BNs by Duering et al. (2013). The model was identified using a tabu search with the Bayesian Gaussian likelihood equivalent as the score applied over a data set of subjects with a genetic small vessel disease causing VCI. Associations and inter-relationships between regional volumes of ischemic lesions in major white matter tracts and processing speed were obtained using a bootstrapping approach.

*B. Supervised classification*. BN classifiers have been applied in several neurodegenerative diseases. Parkinson's disease development prediction through neuroanatomic biomarkers provided by MRI with several BN classifiers were learned by Morales et al. (2013) from subjects in three different stages of the illness: cognitively intact patients, patients with MCI and patients with dementia.

Diciotti et al. (2012) applied naive Bayes to discriminate healthy controls from mild AD patients and patients with MCI from mild AD patients. The predictor variables consisted of subcortical volumes and cortical volumes, cortical thickness and cortical mean curvature extracted from several ROIs. Prediction of disease progress is of great importance to AD researchers, clinicians and patients. Chen et al. (2012b) developed an ensemble of BNs to determine whether or not a subject with MCI will contract AD within a 5-year period based on structural magnetic-resonance and magnetic-resonance spectroscopy data. These variables were used along with age, sex, handedness, education, and mini-mental state examination as potential predictor variables. Zhang et al. (2014) compared the behavior of four classifiers (naive Bayes among them) to automatically distinguish MCI patients from normal controls. The predictor variables corresponded to the cortical thickness of many non-cerebellar ROIs were selected with *t*-tests.

#### 5.4.3. EEG

Unlike other techniques as fMRI, EEG offers a high temporal but low spatial resolution.

*A. Association discovery*. Interactions between brain regions in response to visual stimuli were derived in Song et al. (2009) using healthy subjects who imagined a body part movement based on visual cues. For each subject, a novel model, *time-varying* dynamic BN, was introduced. Thus, the transition model is time dependent, i.e., it is *p*^*t*^(**x**^*t*^|**x**^*t* − 1^). Edge directions come from assuming auto-regressive dynamic BNs and their coefficients are also time dependent. Scalability and the problem of sample scarcity was addressed using a specific score to learn these graphs.

Scalp wake EEG and sleep EEG recordings were used in De la Fuente et al. (2011) jointly with clinical neurologic soft signs and two endocrine tests. The objective was to discover statistical interconnections and interdependences between these variables in borderline personality disorder (BPD) subjects. The contribution of each arc to the global K2 score of the BN was used to measure the degree of interaction between variables.

*B. Supervised classification*. EEG is most often used to diagnose epilepsy, which causes obvious abnormalities in its readings. During a seizure the EEG is characterized by continuous rhythmical activity that has a sudden onset (ictal EEG). During the time between seizures the EEG displays isolated sharp transients or small spikes in some locations of the brain (interictal EEG), which constitute complementary information. Visual inspection of these EEG signals for the presence of seizures is time consuming and often leads to the misdiagnosis of epilepsy. A naive Bayes with continuous distributions approximated by kernels was used in Valenti et al. (2006) to detect interictal spikes isolating them from the baseline EEG activity. Acharya et al. (2011) made a more thorough classification distinguishing between normal (healthy patients), interictal and ictal EEG signals (epileptics). Predictive features were extracted using a non-linear data analysis method called Recurrence Quantification Analysis (RQA). RQA measures are different during the preictal, interictal and ictal stages. A filter feature selection was performed by means of an ANOVA test. A Gaussian naive Bayes classifier and a Gaussian mixture model learned with the EM algorithm were used. Although the latter is an unsupervised technique, the fitted mixture density was presumably used to compare the posterior probabilities of each class and select the MAP.

EEG signals can be used in a brain computer interface (BCI) because they are correlated with mental activities. EEG signals from a set of subjects performing different mental tasks were analyzed in Rezaei et al. (2006). Predictive variables were the (adaptive) autoregressive coefficients of the EEG windows. In order to classify these mental activities from the EEG signals, an HMM and a Gaussian mixture model (as in Acharya et al., 2011) were trained using the EM algorithm. The observed states in the HMMs were the extracted EEG features, also assumed to be generated by a Gaussian mixture model. A common BCI acting as a virtual keyboard is the P300 Speller. Typing speed can be slow since several trials must be averaged to correctly classify responses due to a low signal-to-noise ratio. To speed up the process, Speier et al. (2012) gave the classifier information about the natural language to create a prior belief about the characters to be chosen. A better classifier used prior probabilities for characters from frequency statistics in an English language corpus. Only trigram models were used, that is, *p*(*x*_*t*_|*x*_*t* − 1_, …, *x*_0_) = *p*(*x*_*t*_|*x*_*t* − 1_, *x*_*t* − 2_). Since typing is a sequential process, the same research group improved this model in Speier et al. (2014) with an HMM of a second-order Markov process. The hidden states were the target characters and the EEG signals were the observed variables. The goal was to determine the optimal sequence of target characters given the observed EEG signal with automatic error correction.

Other EEG applications follow. Zhang et al. (2006) designed a system for testing hearing acuity with a general BN containig the class node. Apart from using the BN for classification, this paper is singular because it includes an example of inference. Specifically, a prediction of the class is inferred given an evidence on its parent nodes. There were two different goals in Hausfeld et al. (2012): identify vowels and the speaker who uttered the vowel. Different versions of a Gaussian naive Bayes, always with a binary class (vowel *i* vs. *j* or speaker *l* vs. *h*), were used. Versions differed in the features (EEG voltages) included in the model: in the temporal domain (predefined windows, shifting windows, whole trial period) and in the spatial domain (single channel, multichannel), allowing combined classification analyses. De Vico Fallani et al. (2011) also tackled the problem of person identification with a Gaussian naive Bayes. The EEG signals were recorded during a 1-min resting state with either eyes open or eyes closed (two different problems). The eyes closed resting state yielded better recognition rates.

#### 5.4.4. Others

Electrocorticography (ECoG) or intracranial EEG records cerebral cortex activity with intracranial electrodes placed directly on the brain surface (invasive procedure). ECoG offers high signal-to-noise ratio and high spatiotemporal resolution. Wang et al. (2011) examined the feasibility of an ECoG-based BCI system with four subjects undergoing epilepsy seizure ECoG monitoring and presurgical brain mapping. Features were obtained from the time domain signals.

Scanning electromyography (EMG) records the electrical activity produced by skeletal muscles. Goker et al. (2012) took scanning EMG data from the biceps muscles of healthy subjects and juvenile myoclonic epilepsy (JME) patients to correctly classify them.

Transcranial Doppler (TCD) is a non-invasive ultrasound technology that detects the changes in cerebral blood flow velocity. Recently used for BCI development, TCD-BCI studies have been offline. Lu et al. (2014) implemented an *online* TCD-BCI system to control an onscreen keyboard. User- and session-specific Gaussian selective naive Bayes classifiers were built to discriminate between the activation and rest tasks. Features were chosen using an F-score ranking followed by a wrapper feature selection according to that ranking.

Diffusion tensor imaging (DTI) can reveal abnormalities in white matter fiber structure; it is a standard for white matter disorders. The use of DTI to detect AD dementia requires large samples across multiple sites. Therefore, the effects of different MRI scanners should be accounted for. Dyrba et al. (2013) collected data from many subjects from nine different scanners. A Gaussian selective naive Bayes was used to discriminate between AD patients and controls. Feature (voxels) selection using information gain was necessary because of the high number of voxels. Besides the usual cross-validation for estimating the performance of the methods, an original scanner-specific cross-validation was proposed, where data from each scanner was used as a test set and data from the remaining scanners as a training set.

Ayhan et al. (2013) selected PET scans from Alzheimer's Disease Neuroimaging Initiative (ADNI) project to discern three levels of dementia. Different Gaussian selective naive Bayes were employed, where features were selected with the correlation feature selection filter. Huang et al. (2011) used PET images again from the ADNI project, with AD patients and controls. A Gaussian BN was built for each group in order to find connectivity differences between them. The total number of arcs in both networks was counted to confirm loss of connectivity in AD. Arcs were also counted in each of the four lobes and between each pair of lobes. An arc from region *X* to *Y* was interpreted as *X* having a dominant role in the communication with *Y*, though this is an overinterpretation (see the fMRI section above). But, interestingly, BN learning included two penalties. One was an L1-regularization (as in Schmidt et al., 2007; Vidaurre et al., 2010) to output sparse graphs and another ensured the graph was a DAG.

#### 5.4.5. Multimodal neuroimaging

The next works use more than one technique to maximize neuronal information.

*A. Association discovery*. Plis et al. (2010) presented an integrated analysis of fMRI and MEG. The high temporal resolution of MEG and the full brain coverage with high spatial resolution of fMRI without a spatial inverse problem are complementary and both are expected to jointly improve neural activity estimation. MEG and fMRI data (observed variables *M* and *B*) were tied together in a dynamic BN through a state variable *R* that represents neural activity in a single ROI (hidden variable). MEG and fMRI have different sampling frequencies. Therefore, the time slices in the dynamic BN were the (more detailed) MEG sampling time periods, also including nodes corresponding to unobserved BOLD time points. Arcs from *R* to *M* and from *R* to *B* represented, respectively, forward models used to estimate MEG measurements and BOLD responses, conditioned to the neural activity of the ROI. Since general continuous densities were assumed and the forward models were non-linear, a sequential Monte Carlo method called particle filtering was used to estimate the posterior distribution of *R*. The same research group investigated the different connectivity patterns produced with fMRI and MEG data in Plis et al. (2011). Since the goal was to estimate connectivity, this time the model was very different: a (static) BN with discretized random variables and a high number of ROIs. The structural differences inferred from either modality were summarized via standard aggregated metrics used in complex networks (in- and out-degree, degree centrality, diameter, average path length, etc.).

*B. Supervised classification*. Classifying glioblastomas vs. solitary brain metastases is challenging because both show similar characteristics on conventional MR examination. To improve diagnostic accuracy Chen et al. (2013) used four imaging modalities: DTI, dynamic susceptibility contrast (DSC) MRI, T1-weighted MR, and fluid attenuation inversion recovery. Variables were selected after applying a Wilcoxon rank-sum test. A general BN was learned using a BDe score and MCMC. The Markov blanket of *C* identified which lesion part and modality provided enough information to accurately predict glioblastomas. It was noted that BNs were able to deal with missing data (due, for example, to recording failures or patient disability). Some meningiomas display an atypical radiological appearance and may resemble metastatic lesions or high-grade gliomas. Svolos et al. (2013) distinguished between the three, atypical meningiomas, glioblastomas multiforme and solitary metastases, using a naive Bayes. Just three variables from DTI and DSC modalities used in Chen et al. (2013) were used again here. Two tumor regions (intratumoral and peritumoral) resulted in two models. A similar study is Tsolaki et al. (2013).

Identifying the experimental methods used in human neuroimaging papers is relevant for grouping meaningfully similar experiments for meta-analysis. An automatic system able to replicate the expert's annotation of multiple labels per abstract is useful for the previous task (Turner et al., 2013). The labels included the experimental stimuli, cognitive paradigms, response types, and other relevant dimensions of the experiments. Predictor variables were extracted from the abstract papers by means of text mining methods. That was a multi-label classification problem, approached by a binary relevance method that used a naive Bayes as base classifier.

## 6. Discussion

Well-grounded on principled probability theory, BNs provide clear semantics and a sound theoretical foundation. BNs are easy to comprehend. They visually illustrate the way in which the different variables are related to each other. Their widespread use by numerous research groups, companies, societies and conferences is remarkable. Models can be built from data and or elicited from experts. BNs can handle continuous, discrete, mixed, and temporal variables. BNs are still applicable when some data are missing. A plethora of amenable both exact and approximate learning and inference algorithms are available^1^.

The generality of this formalism makes BNs useful across a wide variety of domains and circumstances. The aim of this survey was to show the potential functionality of BNs in neuroscience, where they have been little used so far. We found that BNs have been mostly used for supervised classification in problems like categorizing interneurons, decoding cognitive states or discriminating control subjects from neuropathological patients (Parkinson's disease, Alzheimer's disease, schizophrenia, depression, glioma, epilepsy, bipolar disorder, dementia, brain metastasis, glioblastomas). The simplest structures were used, i.e., naive Bayes and Gaussian naive Bayes. Very few other models, like TAN, multinets, ensembles, selective models or kernel-based models, were found. Classifiers with high-order degree interaction between variables (k-dependence, BAN and unrestricted Bayesian classifiers), not found in this survey, could capture more complex relationships. Note also that few works performed feature subset selection, necessary to eliminate irrelevant and redundant variables. However, this is a salient issue in modern neuroscience where data volume is growing exponentially.

For temporal inputs, like electrophysiological data or data from fMRI and EEG experiments, dynamic BNs were frequently used to discover associations between variables, as in connectivity analyses, for both task-based and resting-state data and in healthy and diseased patients. Typically, data were discretized or assumed to be Gaussian distributed. Simple particular cases of dynamic BNs, like HMMs, were relatively popular, whereas, complex time-varying BNs were very seldom used.

This survey also found that neuroscience applications using BNs for inference are rare. Our work on dendritic tree simulation models is one of the few applications. We think that beyond the information rendered by the BN structure to relate the domain variables, conditional probabilities unveil detailed and complementary knowledge to be exploited. Moreover, these initial probabilities that the BN conveys are propagated throughout the network in the light of new observations providing insights, predictions and explanations. In that sense, we envisage that inference facilities have a role to play in neuroscience.

Also, there is hardly any clustering with BNs in neuroscience. This method has two characteristic issues: a probabilistic membership assignment to each of the clusters and a multivariate (Gaussian) density that is factorized according to a DAG. However, most of the probabilistic clusterings did not have any factorization (a dense covariance matrix instead), which is far from the BN spirit. We believe that probabilistic clustering is more accurate than hard clustering, and can lead to competent grouping models based on sparse BNs.

Finally, we should say that BNs and neuroscience have a two-way inter-relationship. BNs may also benefit from the challenging problems posed by neuroscience. For instance, the need to fit densities for angular variables (Bielza et al., 2014) and promote the coexistence of variables of any kind–angular, linear continuous Gaussian and non-Gaussian (Varando et al., 2014), discrete– within the same model calls for new BN designs.

### Conflict of interest statement

The authors are the guest editors of this Research Topic. Therefore the manuscript review process has been managed from the main journal. The authors declare that the research was conducted in the absence of any commercial or financial relationships that could be construed as a potential conflict of interest.

## Footnotes

^1^An updated list of software on BNs is available at http://www.cs.ubc.ca/~murphyk/Software/bnsoft.html and http://www.cs.iit.edu/~mbilgic/classes/fall10/cs595/tools.html
